# Identification of Estrogen Receptor Dimer Selective Ligands Reveals Growth-Inhibitory Effects on Cells That Co-Express ERα and ERβ

**DOI:** 10.1371/journal.pone.0030993

**Published:** 2012-02-07

**Authors:** Emily Powell, Erin Shanle, Ashley Brinkman, Jun Li, Sunduz Keles, Kari B. Wisinski, Wei Huang, Wei Xu

**Affiliations:** 1 McArdle Laboratory for Cancer Research, University of Wisconsin–Madison, Madison, Wisconsin, United States of America; 2 Departments of Statistics and of Biostatistics and Medical Informatics, University of Wisconsin–Madison, Madison, Wisconsin, United States of America; 3 UW Carbone Cancer Center, University of Wisconsin School of Medicine and Public Health, University of Wisconsin–Madison, Madison, Wisconsin, United States of America; 4 Department of Pathology and Laboratory Medicine, University of Wisconsin–Madison, Madison, Wisconsin, United States of America; Oklahoma Medical Research Foundation, United States of America

## Abstract

Estrogens play essential roles in the progression of mammary and prostatic diseases. The transcriptional effects of estrogens are transduced by two estrogen receptors, ERα and ERβ, which elicit opposing roles in regulating proliferation: ERα is proliferative while ERβ is anti-proliferative. Exogenous expression of ERβ in ERα-positive cancer cell lines inhibits cell proliferation in response to estrogen and reduces xenografted tumor growth *in vivo*, suggesting that ERβ might oppose ERα's proliferative effects via formation of ERα/β heterodimers. Despite biochemical and cellular evidence of ERα/β heterodimer formation in cells co-expressing both receptors, the biological roles of the ERα/β heterodimer remain to be elucidated. Here we report the identification of two phytoestrogens that selectively activate ERα/β heterodimers at specific concentrations using a cell-based, two-step high throughput small molecule screen for ER transcriptional activity and ER dimer selectivity. Using ERα/β heterodimer-selective ligands at defined concentrations, we demonstrate that ERα/β heterodimers are growth inhibitory in breast and prostate cells which co-express the two ER isoforms. Furthermore, using Automated Quantitative Analysis (AQUA) to examine nuclear expression of ERα and ERβ in human breast tissue microarrays, we demonstrate that ERα and ERβ are co-expressed in the same cells in breast tumors. The co-expression of ERα and ERβ in the same cells supports the possibility of ERα/β heterodimer formation at physio- and pathological conditions, further suggesting that targeting ERα/β heterodimers might be a novel therapeutic approach to the treatment of cancers which co-express ERα and ERβ.

## Introduction

Estrogens exert their biological effects via interaction with two estrogen receptors (ERs), ERα and ERβ [1], [2]. ERs regulate key physiological functions in the reproductive tract, breast, prostate, bone, brain and the cardiovascular system [1], [2]. In some organs, ERα and ERβ are expressed at similar levels but in different cell types [3]. For example, in the prostate, ERα is predominately expressed in stroma while ERβ is expressed in the epithelium. Both receptors are expressed in normal mammary epithelial cells [4]. Studies with ERα knockout mice (αERKO) demonstrate that ERα is essential for ductal formation and mammary gland development [5]. Although ERβ knockout mice (βERKO) generate mild mammary phenotypes, Ki-67 expression is increased in luminal mammary epithelial cells of βERKO mice [6], suggesting that ERβ may be important for terminal differentiation of mammary epithelial cells. ERα and ERβ are also involved in growth and differentiation of the prostate gland and progression of prostate disease [7], [8]. A recent study showed that stromal ERα promotes prostatic carcinogenesis [9]. Moreover, hyperplasia was observed in the prostates of βERKO mice [10] and ERβ expression was silenced in a subset of malignant human breast and prostate cancers [11], [12], suggesting that ERβ plays protective roles in these diseases.

The classic mechanism through which the ERs modulate gene expression is a cascade of events: ligand binding to ERα or ERβ induces receptor dimerization, either as homodimers (ERα/ERα or ERβ/ERβ) or heterodimers (ERα/ERβ), translocation of dimers to the nucleus, and recognition of Estrogen Response Elements (EREs) on DNA. The target genes activated by these events, and hence the physiological responses, depend on the dimer pair activated by the ligand. Indeed, several studies have shown that ERα and ERβ exhibit opposing roles in cellular proliferation and apoptosis, with ERα inducing the transcription of pro-proliferative and anti-apoptotic target genes, and ERβ being anti-proliferative and pro-apoptotic [13], [14], [15]. In accordance with this notion, target gene studies reveal that ERα and ERβ may have distinct biological functions; it is believed that ERα promotes cell growth, while ERβ inhibits it in breast and prostate cancer cells [11], [14], [16], [17], [18], [19]. It has thus been deduced that the role of the ERα/α homodimer is to accelerate cellular proliferation, thus lending to carcinogenesis and tumor progression, while conversely the transcriptional activation from ERβ/β homodimers is thought to be protective against hormone-dependent diseases including breast and prostate cancers [13], [14], [15].

ERβ has well known growth modulatory activity in ERα-positive breast cancer cells. Compared with tumors expressing ERα alone, the co-expression of ERβ has been correlated with a more favorable prognosis [20] and decreased biological aggressiveness [11], [21], [22], [23], [24]. Moreover, ERβ has been shown to modulate the proliferative actions of estrogens when co-expressed with ERα [13], [19], [25], [26] and can be considered an endogenous partial dominant negative receptor [27], [28]. ERβ is thought to counteract the stimulatory effects of ERα through heterodimerization of the two receptors [29], [30]. Indeed, these heterodimers have been shown to form and maintain function [31], and they have been suggested to be responsible for the activation of target genes which are distinct from those induced by either homodimer [32], [33]. The co-expression of ERβ with ERα results in reduced ERα-mediated proliferation and invasion of breast cancer cells [11], [16], [17], [18], [19], at least in part due to ERβ's inhibition of ERα selective target gene expression. Furthermore, in the ERα/ERβ-positive mouse mammary epithelial cell line HC11, ERα drives cellular proliferation whereas ERβ contributes to growth inhibition and apoptosis in response to 17β-estradiol; (E2); the loss of ERβ in this cell line results in cellular transformation [14]. Thus, the ERα∶ERβ ratio determines whether E2 will induce cellular proliferation. Despite the fact that the ERα/β heterodimer has been proposed to have a biological role that is unique from that of either homodimer, the biological function of these heterodimers *in vivo* has until now remained elusive, at least in part due to the heterogeneous population of dimers existent upon the co-expression of ERα and ERβ and the lack of heterodimer-specific compounds to elucidate their functions.

**Figure 1:**
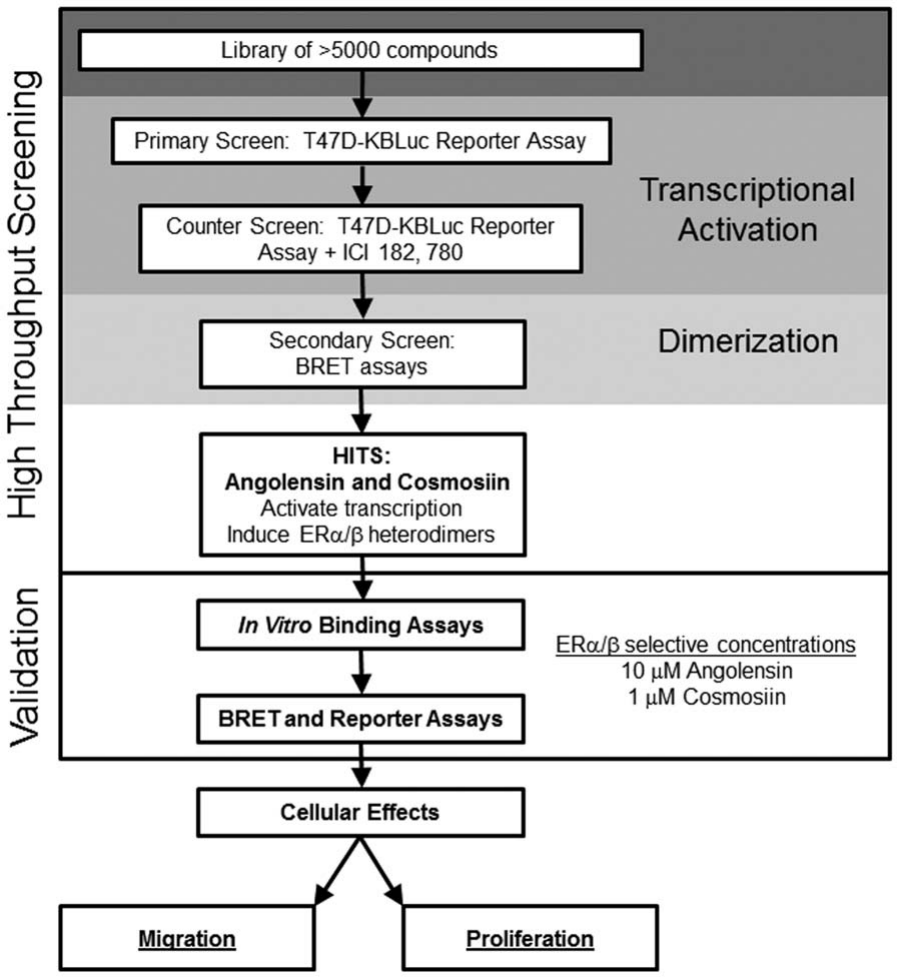
Flow scheme of high throughput screening and characterization of compounds with selectivity for ERα/ERβ heterodimers. A library of >5200 small molecules was screened ER transcriptional activity using T47D-KBLuc cells. Molecules with transcriptional activity were then screened for ERα/α, ERα/β, or ER β/β dimerization potential using BRET assays. Two phytoestrogens, angolensin and cosmosiin, were identified as ER dimer selective ligands. These molecules were validated using *in vitro* binding assays and BRET and ERE-luciferase reporter assays. Heterodimer selective concentrations were identified as 10 µM angolensin and 1 µM cosmosiin. The cellular effects of these two heterodimer-selective concentrations were characterized using cell migration and proliferation assays.

To circumvent this issue, the identification of ERα/β heterodimer-selective ligands that activate the transcriptional effects of ERα/β heterodimers, but not that of either homodimer, were sought in order to shed light upon the transcriptional outcomes and biological roles of these heterodimers. To this end, a multi-step high throughput small molecule screen for ER transcriptional activation and dimer selectivity was developed (Figure 1). This screening resulted in the identification of two phytoestrogens that are transcriptionally active and ERα/β heterodimer-selective at specific concentrations. These compounds were rigorously characterized for their biological activity in cell-based assays (Figure 1). The results of these studies suggest that the ERα/β heterodimer exerts growth inhibitory effects in breast and prostate epithelial cells. These compounds may serve not only as tools for deciphering the biological functions of the ERα/β heterodimer, but also potentially as a means for therapeutically targeting ERα/β heterodimers in hormone-dependent diseases including breast and prostate cancers.

## Results

### Characterization of Lead Compounds Cosmosiin and Angolensin Using Bioluminescence Resonance Energy Transfer (BRET) and Reporter Assays

**Figure 2:**
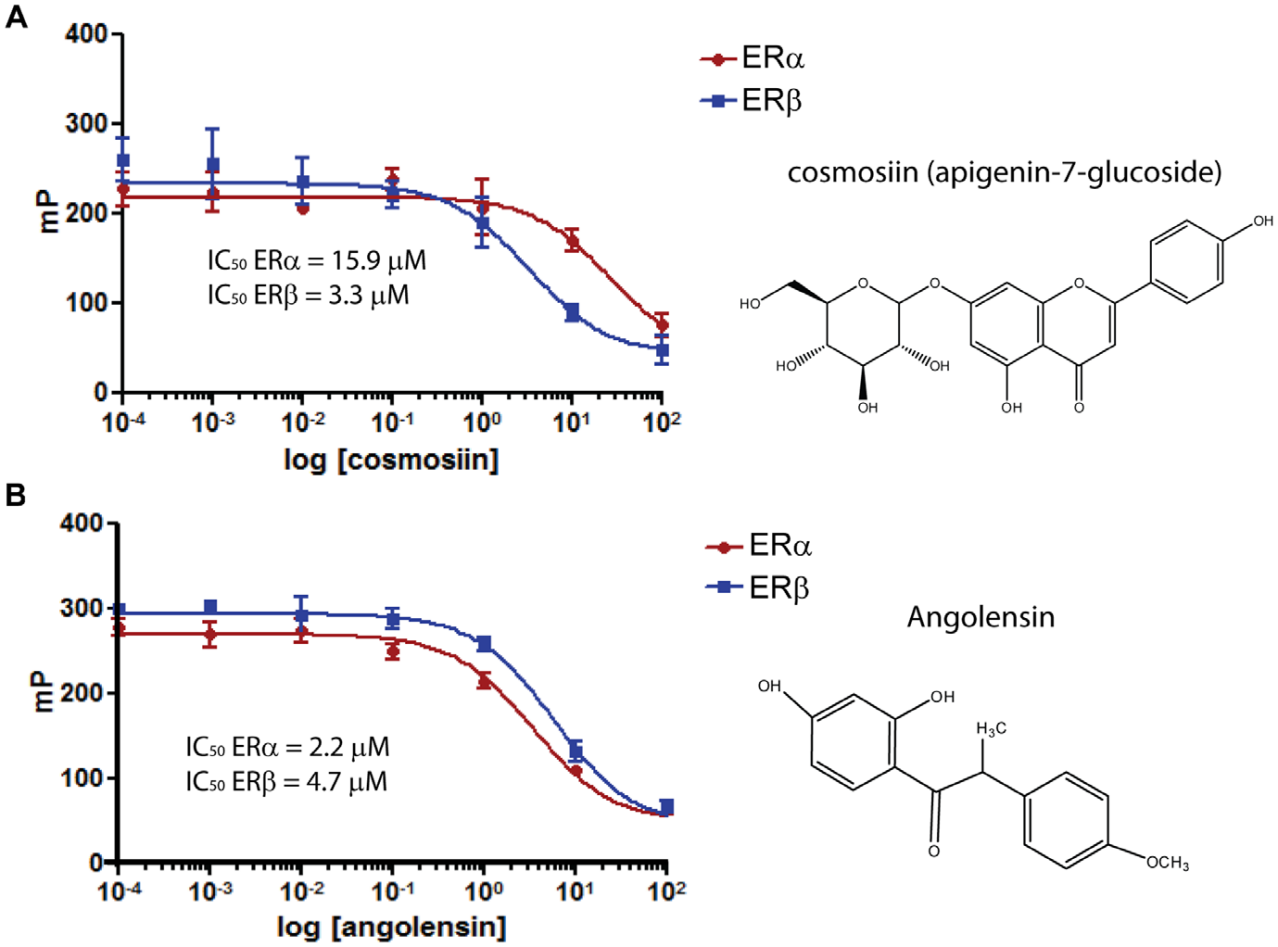
Fluorescence polarization competition binding assays for ERα and ERβ. Cosmosiin (A) and angolensin (B) bind to recombinant ERα and ERβ with µM affinities.

We developed two-step high throughput screening (HTS) for identification of ER dimer-selective ligands (unpublished). The primary screening and counter-screening in the presence of the antagonist ICI 182,780 (Fulvestrant) for ER-specific transcriptional activity was performed in T47D-KBLuc as described in the Methods section. ER dimer selectivity of the primary hits was assessed in secondary HTS BRET assays as described in the Methods section and in [34]. Several compounds with dimer selectivity were identified after performing two-step HTS on >5200 compounds at the UWCCC Small Molecule Screening Facility (unpublished results). Two phytoestrogens, cosmosiin (apigenin-7-glucoside) and angolensin (R) (Fig. 2), were identified in HTS as ER dimer selective ligands. Angolensin exists in two enantiomeric forms; only the R form was identified and used in this study and is thus abbreviated as angolensin hereafter. To determine if they bind the same ligand binding pocket as 17β-estradiol and to measure their binding affinity to recombinant ERs, we employed *in vitro* Fluorescence Polarization (FP) competition binding assays [35]. The IC_50_ values for cosmosiin binding to ERα and ERβ were 15.9 µM and 3.3 µM, respectively (Fig. 2A). The IC_50_ values for angolensin binding to ERα and ERβ were 2.2 µM and 4.7 µM, respectively (Fig. 2B).

**Figure 3:**
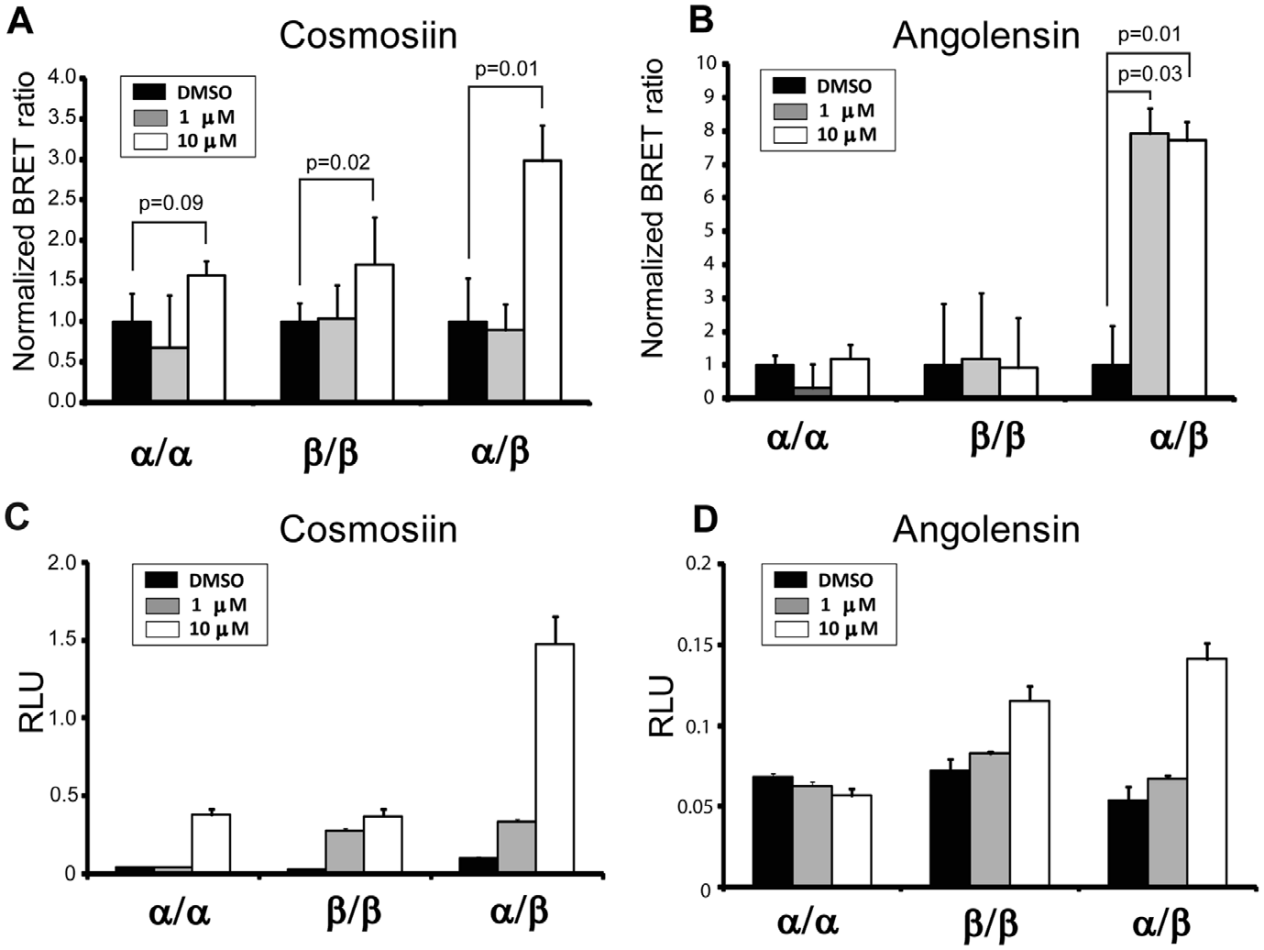
The dimer selectivity for cosmosiin and angolensin was demonstrated in dose-response BRET assays (A and B) and reporter assays (C and D) in HEK293 cells. ER dimer-specific BRET assays were performed over a range of compound concentrations of cosmosiin (A) and angolensin (B). HEK293T ERE-luciferase transcriptional assays reveal each compound's ability to transcriptionally activate various dimer pairs (C and D). ERα alone, ERβ alone, or ERα+ERβ was transfected along with an ERE-luciferase element in order to test the ability of cosmosiin (C) and angolensin (D) to transcriptionally activate these various ER dimer pairs. RLU, relative luciferase units. Error bars represent standard deviations from the mean of triplicate samples. In BRET (A), p values indicate all pairs with statistical significance by the Student's T-Test.

The ER dimer selectivity was validated in BRET and reporter assays in ER-negative HEK293 cells as described [35]. While cosmosiin exhibits preference for inducing both ERβ/β homodimers and ERα/β heterodimers (Fig. 3A), angolensin exhibits ERα/β heterodimer selectivity (Fig. 3B). Neither compound shows preference for inducing ERα/α homodimers. Because the lower limit of detection for these compounds was 1 µM, concentrations lower than 1 µM are not shown in this figure, although they were tested in a range from 1 nM to 10 µM; below 1 µM, the BRET ratios were the same as vehicle-treated. Furthermore, the ability of these lead compounds to induce the transcriptional activity of ERα alone, ERβ alone, or ERα in combination with ERβ was tested at a range of concentrations using the HEK293 ERE-luciferase reporter assays (Fig. 3C and 3D). Although these reporter assays do not directly examine ERα/β heterodimerization, the condition in which ERα and ERβ are cotransfected can be compared with each receptor transfected alone.

As shown in Figure 3B, BRET assays reveal that angolensin is capable of efficiently inducing the *formation* of ERα/β heterodimers at 1 µM and 10 µM, while not inducing ERα/α or ERβ/β homodimers. ERα/β heterodimerization appears to be favored in the presence of angolensin , and in the condition in which ERα and ERβ are co-transfected for luciferase reporter assays, the highest fold induction of transcriptional activity relative to DMSO vehicle is observed (Fig. 3D). Thus, angolensin (R) appears to be an ERα/β heterodimer-selective ligand at 10 µM. Cosmosiin appears to be less selective in terms of its ability to induce ERα/β heterodimers, as ERβ/β homodimers are also induced in BRET assays; however, ERα/α homodimers are not induced by cosmosiin (Fig. 3A). Cosmosiin at 1 µM appears to transcriptionally activate ERβ/β homodimers and ERα/β heterodimers (Fig. 3C). At 10 µM cosmosiin, while ERα/α and ERβ/β homodimers were slightly activated, co-transfecting ERβ with ERα exhibited much stronger transcriptional activity (Fig. 3C). Thus, cosmosiin appears to be ERβ/β homodimer- and ERα/β heterodimer-selective at 1 µM.

The transcriptional activity of ERα/α homodimers treated with 10 µM cosmosiin is despite the finding that the BRET assay does not show statistically significant ERα/α homodimerization (Fig. 3A). The most likely explanation for this discrepancy is differences in sensitivity between BRET and the luciferase reporter assays. These BRET assays and luciferase reporter assays are performed under different conditions and measure different signal outputs: BRET captures a single moment in time in which ERα and ERβ may or may not be dimerized. This moment in time was observed after 1 hour incubation with ligand. Conversely, the luciferase reporter assay measures an accumulation of transcriptional output signal (the transcribed luciferase protein) over 18–24 hours. Consequently, the dimerization ratios obtained via the BRET assay do not always completely agree with the transcriptional profiles obtained in the luciferase reporter assays for a given ligand. Therefore, it is important to consider the direct dimerization of ERα and ERβ in conjunction with the transcriptional output of these diverse dimer pairs.

### Selection and generation of cell lines expressing different amounts of ERα and ERβ

**Figure 4:**
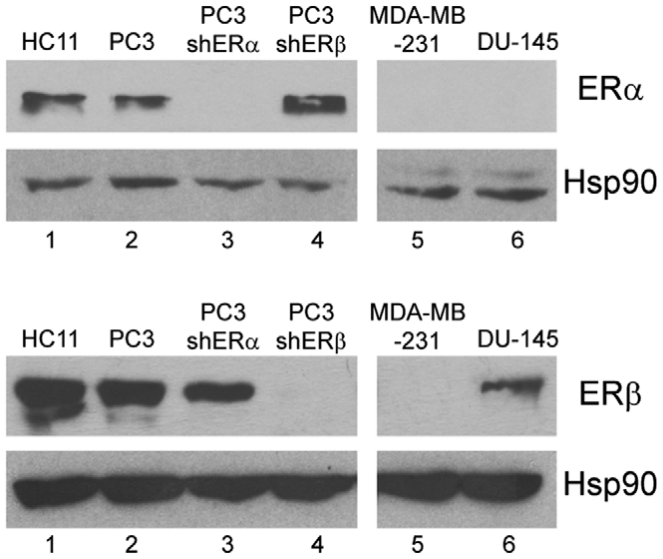
Determination of relative expression levels of ERα and ERβ in various cell lines. Western blotting analyses of ERα and ERβ expression in HC11 (lane 1), PC3 (lane 2), PC3shERα (lane 3), PC3shERβ (lane 4), MDA-MB-231 (lane 5), and DU145 (lane 6).

In order to characterize the cellular effects of cosmosiin and angolensin, we surveyed a variety of breast and prostate cell lines for co-expression of ERα and ERβ. As shown in Fig. 4, the non-tumorigenic mammary epithelial cell HC11 and prostate cancer cell line PC3 were found to express both receptors (Lanes 1 and 2) as reported by others [14], [36]; in contrast, DU-145 expresses only ERβ [36] (lane 6) and MDA-MB-231 is negative for both ERα and ERβ (lane 5). To delineate the functions of ERα/β heterodimers, we knocked down ERα and ERβ transcript levels in PC3 cells by means of stable transfection with specific shRNA plasmids targeting ERα and ERβ, respectively. Western blotting results showed that ERα is selectively silenced in PC3-shERα cells and ERβ is selectively silenced in PC3-shERβ cells (Fig. 4A, lanes 3 and 4). The silencing of one ER did not influence the expression of the other. All of these characterized cell lines were subsequently used for determination of compounds' cellular effects.

### Cosmosiin and angolensin inhibit cell motility and migration but not apoptosis in PC3

**Figure 5:**
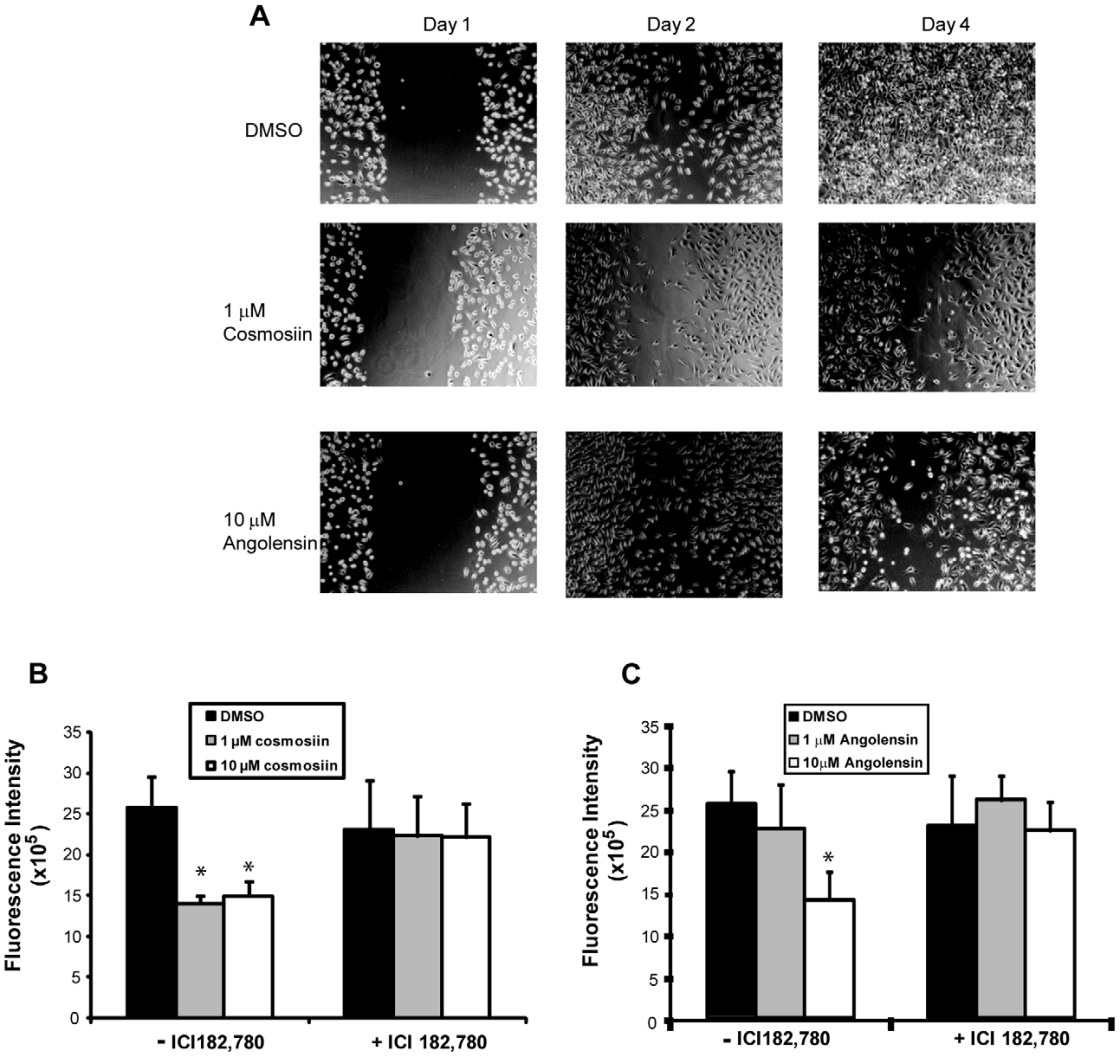
Cosmosiin and angolensin inhibit PC3 cell motility and migration. (A) Wound healing assays showing the effect of 1 µM cosmosiin and 10 µM angolensin on ERα,β-positive PC3 cells. Vehicle (DMSO) treatment resulted in cell motility to fill the wound (*top panels*) that was inhibited by 1 µM cosmosiin (*middle panels*) and 10 µM angolensin (*bottom panels*). (B) Transwell migration assays measured the ability of cosmosiin (B) and angolensin (C) to inhibit cellular migration of PC3 cells toward a chemoattractant. Cosmosiin (B) and angolensin (C) decreased the ability of PC3 cells to migrate through the pore, and this decreased ability was ablated by the antagonist ICI 182,780 at 100 nM.

In order to examine the influences of these ERα/β heterodimer-activating compounds on cell migration, wound healing assays were employed using migratory PC3 cells. This assay gives a qualitative measure of a compound's ability to inhibit cell migration. For these assays, 1 µM cosmosiin and 10 µM angolensin were utilized because these are the concentrations at which ERα/β heterodimers are most highly selectively induced by each respective compound. As shown in Figure 5A, the vehicle DMSO (0.1%) was unable to inhibit the migration of PC3 cells in scratch wound healing assays: cells can be seen infiltrating the wound 24 hours after scraping, and the wounds are completely filled 72 hours after scraping. Conversely, both 10 µM angolensin and 1 µM cosmosiin are able to inhibit the ability of PC3 cells to infiltrate the wounds, indicating that these compounds can hinder cell motility.

To quantitatively measure the ability of cosmosiin and angolensin to inhibit cell migration, transwell assays were employed. Figure 5C shows that 10 µM angolensin can inhibit the ability of PC3 cells to migrate through the pore, and this inhibition of migration is ablated by the ER antagonist ICI 182,780. 1 µM angolensin, a concentration at which ERα/β heterodimers are not transcriptionally active (Fig. 3D), has a negligible effect on cell migration. Both 1 µM and 10 µM cosmosiin can inhibit cell migration through the pore, and this inhibition of migration is ablated by ICI 182,780 (Fig. 5B). These results are recapitulated when the transwell is coated with matrigel (data not shown), indicating that in addition to dampening the ability of PC3 cells to migrate, these compounds are able to dampen the ability of PC3 cells to invade.

The abilities of these lead compounds to influence apoptosis in PC3 cells were next evaluated using caspase 3/7 assays. PC3 cells were incubated with the indicated concentrations of DMSO vehicle (0.1%), the indicated concentrations of cosmosiin or angolensin (Fig. S1A and S1B), or the positive control cisplatin (10 µg/mL) for 24, 48, and 72 hours. Cisplatin did not activate the caspases 3/7 pathway at 24 hours and 48 hours (data not shown); only at 72 hours was a weak induction of the caspases 3/7 observed (Fig. S1). At no time point did these compounds reveal any activation of the caspase 3/7 pathway. Thus, it appears that cosmosiin and angolensin are not strong inducers of apoptosis, at least through the caspase 3/7 pathway.

### Determination of the growth effects of compounds in PC3, PC3-shERα, PC3-shERβ cells

To determine if these compounds also inhibit cell proliferation in addition to migration, MTT assays were employed. This assay measures mitochondrial activity when yellow MTT (3-(4,5-Dimethylthiazol-2-yl)-2,5-diphenyltetrazolium bromide) is reduced to its purple formazan metabolic product [37]. Thus, the ability of a cell to metabolize MTT to formazan is correlated to its metabolic activity and cellular growth. To show that PC3 cells express functional ERs and that E2's cellular effects are ER-dependent, we compared E2's growth effects in PC3, PC3-shERα, PC3-shERβ cell lines. As shown in other ERα and ERβ co-expressing cell lines [14], E2 exhibits no effects in proliferation of PC3 (Fig. S2A). However, when ERβ expression was blocked, E2 induced proliferation (Fig. S2C) and E2's proliferative effects were completely abrogated by the pure ER antagonist ICI 182,780 and the ERα selective antagonist MPP dihydrochloride (Fig. S2C, *middle* and *right* panels). This result recapitulates the previous finding in HC11 mammary epithelial cells that ERα drives proliferation in response to E2 [14]. It appears that silencing ERβ in PC3 cells causes the cells to respond to E2 with increased proliferation, similar to breast cancer cells [19], [28]. In contrast to HC11 where ERβ is growth inhibitory, knockdown of ERα did not result in E2-dependent growth inhibition (Fig. S2B). The discrepancy might be due to cell line specific effects.

**Figure 6:**
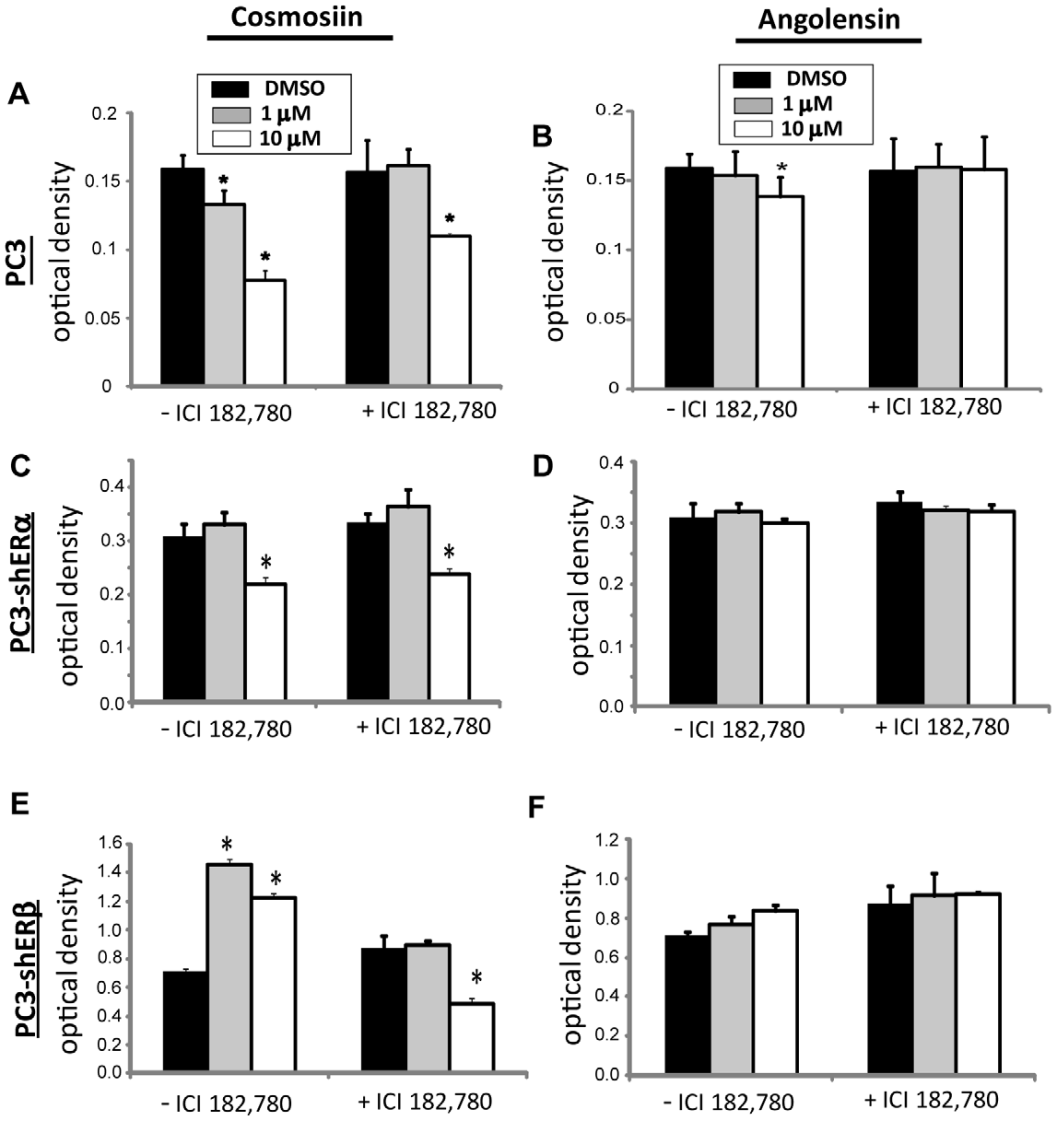
ER dimer-selective compounds influence cell growth in an ER-dependent and dose-dependent manner. Cosmosiin (A) and angolensin (B) decrease the growth of PC3 prostate cancer cells in a dose-dependent manner. These decreases are ER-specific for 1 µM cosmosiin and 10 µM angolensin, since the growth decreases are ablated by the ER antagonist ICI 182,780. These ER-specific effects by 1 µM cosmosiin (C) and 10 µM angolensin (D) are lost with the silencing of ERα in PC3-shERα cells, while ER non-specific effects due to 10 µM cosmosiin are retained. Silencing ERβ in PC3-shERβ results in cosmosiin-dependent increases in cell growth (E) that are ablated in the presence of the antagonist, and furthermore, when ERs are antagonized, ER non-specific growth inhibition in PC3-shERβ is retained. Angolensin has no statistically significant effects in PC3-shERβ (F). Error bars represent standard deviations from the mean of triplicate samples. * indicates statistical significance by the Student's T-Test.

The cellular effects of cosmosiin and angolensin were determined in PC3, PC3-shERα or PC3-shERβ cells at concentrations that display ER selectivity. As shown in Figure 6, both 1 µM and 10 µM cosmosiin (Fig. 6A) and 10 µM angolensin (Fig. 6B) were able to inhibit the growth of PC3 cells compared to the vehicle DMSO. The inhibition of growth due to 10 µM angolensin was ablated by the antagonists ICI 182,780, indicating that this response is ER-specific. The inhibition of growth by 1 µM cosmosiin was also ER-specific. However, the inhibition of growth by 10 µM cosmosiin was not ablated by the antagonist, indicating that this response is not ER-specific in PC3 cells and is likely due to off-target effects or non-genomic ER signaling. Cell counting and viability assays with Trypan blue staining ruled out the possibility of general cytotoxicity due to these compounds (Fig. S3A, S3B).

In PC3-shERα cells, ERβ is the only functional ER present; thus, ERβ/β homodimers are the only ER dimers capable of forming and activating transcription. The growth inhibition observed by 1 µM cosmosiin (Fig. 6C) and 10 µM angolensin (Fig. 6D) in the parent PC3 cells is ablated with the loss of ERα in PC3-shERα cells. The addition of the ER antagonist ICI 182,780 had no effect on this cell line in the presence of 1 µM cosmosiin and 10 µM angolensin compared to these ligands alone. However, 10 µM cosmosiin was still able to inhibit the growth of these PC3-shERα cells in both the absence and presence of the ER antagonist ICI 182,780 (Fig. 6C). Thus, 10 µM cosmosiin is confirmed to have off-target, ER non-specific influences on growth regulation.

In PC3-shERβ cells, ERα is the only functional ER present; thus, ERα/α homodimers are the only ER dimers capable of forming and activating transcription. As shown in Figure 6F, angolensin has a negligible effect in this cell line, and treatment with the ER antagonist ICI 182,780 completely ablates any growth effects observed in the presence of this compound. This finding is consistent with angolensin's high degree of ERα/β heterodimer selectivity. However, contrary to observations in PC3 cells and PC3shERα cells, cosmosiin increases the growth of PC3-shERβ cells at both 1 µM and 10 µM (Fig. 6E). The transcriptional activation of ERα/α homodimers is induced with 10 µM cosmosiin in HEK293 ERE-luciferase assays (Fig. 3C), which is in keeping with its ability to increase the growth of PC3-shERβ cells at this concentration. However, the increase in growth due to 1 µM cosmosiin is not predicted by the HEK293 ERE-luciferase assay (Fig. 3C). These data were confirmed with cell counting and viability assays with Trypan blue staining (data not shown). These growth increases in PC3-shERβ cells due to 1 µM cosmosiin are ablated by the antagonist ICI 182,780 (Fig. 6E), suggesting that these growth increases are due to ERα/α homodimers. Intriguingly, treatment with these antagonists in the presence of 10 µM cosmosiin not only ablates the growth increases observed at this concentration, but actually results in decreased growth (Fig. 6E). This inhibited growth in PC3-shERβ cells when ERα is antagonized may be explained by the off-target effects mediated by this compound: when ERα is the only ER present, it is not damped by heterodimerization with ERβ and is instead able to bind cosmosiin to increase cellular growth; however, when ERα is antagonized in this cell line, cosmosiin is free to mediate its off-target growth inhibitory effects, resulting in decreased growth.

The ERα/β heterodimers were found to be growth inhibitory using PC3 derived cell lines and ERα/β heterodimer-selective ligands at concentrations determined to be heterodimer-selective. The effects of ERα/β heterodimer-selective ligands in PC3 cells suggest that 1 µM cosmosiin and 10 µM angolensin are responsible for mediating the physiological responses of ERα/β heterodimers on a cellular level since loss of either ERα or ERβ abrogates growth inhibition at these concentrations (Fig. 6C–F). 10 µM cosmosiin mediates growth inhibitory effects via ERβ/β homodimerization and off-target effects when ERα is lost, and both concentrations of cosmosiin increase growth via ERα homodimers when ERβ is lost. Therefore, the expression levels of ERs appear to be important to the physiological outcome of these ligands at cellular levels.

### The growth effects of cosmosiin and angolensin on additional cell lines with differing ERα∶ERβ expression ratios

The differing cellular effects in PC3, PC3-shERα, and PC3-shERβ suggest that the ratio of ERα∶ERβ may be a determinant for the ability of these dimer-selective ligands to act in a proliferative or anti-proliferative manner. To address this, growth and viability assays in several cell lines with differing expression levels of ERα and ERβ were compared. HC11 is a normal mouse mammary cell line that expresses both ERα and ERβ (Fig. 4A and [14]). As shown in Fig. S3, cosmosiin and angolensin are both able to inhibit the growth of this cell line. Specifically, 1 µM angolensin, a concentration at which ERα/β heterodimers are not predicted to be activated (Figures 3B, D) has no effect on the growth of this cell line, whereas 10 µM angolensin inhibits HC11's growth by ∼10% compared to the vehicle DMSO (Fig. S3D), and this inhibition is ablated by the antagonist ICI 182,780, which suggests that this inhibition is ER-specific. Cosmosiin is also able to inhibit the growth of HC11 cells at 1 µM and 10 µM (Fig. S3C). The ∼15% inhibition of growth resulting from 1 µM cosmosiin treatment is ablated by the antagonist ICI 182,780, indicating that this response is ER-specific. 10 µM cosmosiin inhibits the growth of HC11 cells by ∼25% compared to the vehicle DMSO, and this response is not completely ablated by the antagonist ICI 182,780, indicating that the inhibition of proliferation by 10 µM cosmosiin is not ER-specific in agreement with earlier findings (Fig. 6A and 6D). Cell counting and viability assays with Trypan blue staining confirmed these findings of growth inhibition and indicated that they were not due to general cytotoxicity (Fig. S3A and S3B). The growth inhibitory effects of 1 µM cosmosiin and 10 µM angolensin in HC11 cells support the notion that ERα/β heterodimers are growth inhibitory. Furthermore, we examined the compounds' effects on ERα−/ERβ− cell line MDA-MB-231 and ERα−/ERβ+ DU-145. Neither compound has any effect on cell growth at all tested concentrations in MDA-MB-231 breast cancer cells (Fig. S4) nor DU-145 prostate cancer cells (Fig. S5). This result suggests that the growth effects exerted by compounds are ERα and ERβ-dependent in breast and prostate epithelial cells. This conclusion is supported by the findings that growth effects elicited by 1 µM cosmosiin and 10 µM angolensin could be completely antagonized by ER antagonist in PC3, PC3-shERα, and PC3-shERβ cells (Fig. 6).

### Nuclear co-localization of ERα and ERβ in human breast tumor specimen

**Figure 7:**
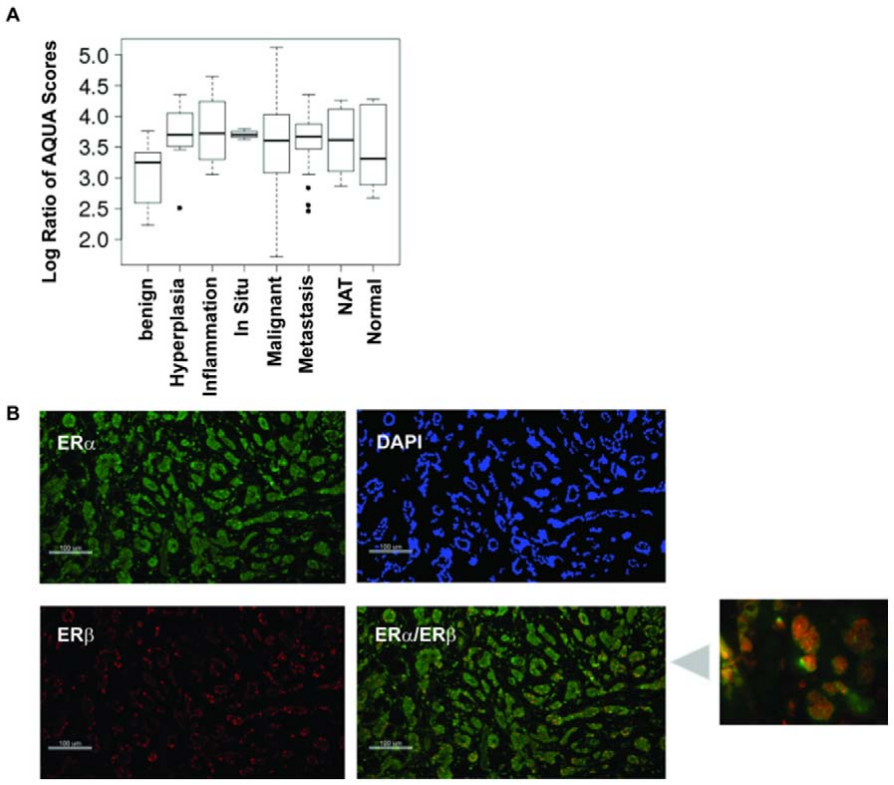
Automated quantitative measurement of ERα and ERβ expression in breast cancer tissue microarrays. (A) Tissue microarray analysis with the AQUA® technology shows that the ERα∶ERβ ratio increases from benign samples throughout various stages of malignancy. Pairwise two-sample t-tests between the benign and malignant samples showed a statistically significant difference (p-values<0,01). (B) AQUA® analysis indicates that ERα and ERβ colocalize to the nucleus within the same cell in human breast tumors. Figures are shown at 200× magnification, and scale bars are present in the lower left corners of each image. The blow-up picture is an amplified section of AQUA staining showing co-localization of ERα and ERβ to the nucleus. * indicates statistical significance by the Student's T-Test. NAT = cancer adjacent normal breast tissue.

Our studies indicate that cosmosiin and angolensin could be therapeutically useful for inhibiting the growth of breast cancer cells that co-express ERα and ERβ. Although previous studies have shown 60% of ERα-positive breast tumors express ERβ [11], [21], in order for ERα/β heterodimerization to occur, ERα and ERβ must be co-expressed in the same cell. To investigate the co-expression of ERα and ERβ in breast tumor samples, we analyzed a breast cancer tissue microarray (TMA) using the quantitative immunofluorescence AQUA® technology (HistoRx) that allows the quantitative measurement of proteins of interest within subcellular location of tissue samples by calculation of an AQUA® score. Such precision is not possible with conventional testing methods, such as standard immunohistochemistry (IHC). This TMA was purchased from US Biomax (BR2082) and contained 32 cases of metastatic carcinoma, 68 cases of invasive ductal carcinoma, 22 cases of invasive lobular carcinoma, 22 cases of intraductal carcinoma, 4 cases each of squamous cell carcinoma and lobular carcinoma in situ, 8 cases of fibroadenoma, 16 cases each of hyperplasia and inflammation, 10 cases of cancer adjacent normal breast tissue (NAT) and 6 cases of normal tissue. A total of 208 cores were analyzed for nuclear ERα and ERβ intensity with DAPI and β-actin staining as references. As shown in Fig. 7A, the ERα/ERβ ratio increases throughout the stages of carcinogenesis and progression. Pairwise analysis with two-sample t-tests of benign tissue versus hyperplasia (p-value = 0.0039) and versus carcinoma (in situ, inflammation, metastatic, and malignant cases with p-values 0.0092, 0.0035, 0.0042, respectively) indicate that this ratio is significantly higher in cases of hyperplasia and carcinoma compared to benign tissue. Figure 7B shows that ERα and ERβ colocalize within the nucleus of the same cell in tissue samples. Overlaying the high resolution images (Fig. 7B, *right*) for ERα staining with those for ERβ staining shows that ERα and ERβ co-localize to the same spots within the same nucleus, rendering the possibility that ERα/β heterodimerization is feasible in these tissues.

## Discussion

While the roles of ERα and ERβ in hormone-dependent diseases such as breast and prostate cancers are becoming increasingly elucidated, with ERα having a proliferative and ERβ having an anti-proliferative role, the mechanism by which these two receptors interact with each other in both normal and diseased states has remained elusive. Because the co-expression of ERβ along with ERα dampens the proliferative action of ERα, direct interaction of ERα and ERβ is thought to convey growth inhibitory effects, and the ERα/β heterodimer has been proposed to activate target genes mediating these anti-proliferative effects [38], [39]. However, the heterogeneous population of dimer pairs present when ERα and ERβ are co-expressed and the lack of full length heterodimerized ER structures prevent a clear understanding of their biological function. Thus, in order to shed light upon the biological action of these ERα/β heterodimers, we sought to identify small molecule ligands capable of specifically inducing heterodimers while not inducing ERα/α homodimers or ERβ/β homodimers with the rationale that these ligands could be used to decipher the biological action of ERα/β heterodimers.

The BRET technology developed in our lab [34], [35] allowed the examination of each ER dimer pair (ERα/α homodimers, ERβ/β homodimers, and ERα/β heterodimers) in isolation. This segregation was especially essential in the case of the ERα/β heterodimer, as the co-expression of ERα and ERβ leads to the formation of all three dimer forms and prevents separation of the action of each individual dimer pair as they function in concert *in vivo*. However, the BRET assay allows the examination of ERα/β without observing homodimer formation. Specifically, we have previously shown that two phytoestrogens, genistein and liquiritigenin, preferentially induce different ER dimers [34]. Liquiritigenin selectively induced formation of ERβ/β homodimers and ERα/β heterodimers but not ERα/α homodimers at 1 µM [34], which provides proof-of-principle that small molecule compounds which preferentially induce ERα/β heterodimers over ERα/α homodimers do indeed exist. We had further shown that BRET assays can be optimized for HTS [35]. The goal of secondary HTS BRET screening in this study was to find a compound with similar characteristics to liquiritigenin but with greater ERα/β heterodimer selectivity. If a library compound was able to induce ERα/β heterodimerization while not inducing ER homodimerization, the ligand could be used in biological systems to determine the function of these heterodimers with minimal interference from either homodimer.

Two lead compounds were successfully identified in BRET screening. The two lead compounds are flavonoids, a group of potentially chemoprotective compounds widely distributed in fruit, vegetables, and beverages of plant origin including tea and wine, and have similar structures that consist of two phenolic benzene rings linked to a heterocyclic pyre or pyrone [40]. Isoflavones represent an important group of phytoestrogens and are found mainly in plants belonging to the Leguminosae family. Angolensin (*Trifolium pretense*, 2′,4′-dihydroxy-4″-methoxy-α-methyldeoxybenzoin, 1-(2,4-dihydroxyphenyl)-2-(4-methoxyphenyl)propan-1-one; CAS 642-39-7), is an isoflavone that was first isolated from the wood of *Pterocarpus angolensis* and later from the wood and bark of *Pterocarpus indicus*. Angolensin is a metabolite of Biochanin A and formononetin, which are present in red clover [41], [42]. Dietary supplements manufactured from red clover are widely marketed to provide beneficial health effects of isoflavones without dietary changes. Specifically, red clover supplements are often consumed for the purported alleviation of post-menopausal symptoms. Cosmosiin (apigenin 7-O-beta-glucoside; apigenin-7-D-glucoside; apigenin-7-O-beta-D-glucopyranoside; apigenin-7-glucoside; cosmetin, Cosmosiine, Apigetrin,5-hydroxy-2-(4-hydroxyphenyl)-7-[(2S,3R,4S,5S,6R)-3,4,5-trihydroxy-6-(hydroxymethyl)oxan-2-yl]oxychromen-4-one; CAS 578-74-5) is a flavonoid present in chamomile flowers which are used pharmaceutically and cosmetically for their anti-spasmodic, anti-inflammatory and antimicrobial properties and also as a natural hair dye and fragrance. Cosmosiin has also been isolated from *Veratrum grandiflorum* (white hellebore) and *Kummerowia striata* (Korean clover). Cosmosiin has been shown to exhibit anti-inflammatory properties [43] and has been shown to exhibit HIV anti-viral properties [44], although it has not received FDA approval for these purposes. The direct binding of angolensin and cosmosiin to the E2-binding pocket of ERs are observed (Fig. 2). To our knowledge, this is the first demonstration of cosmosiin as an estrogenic compound. Furthermore, we validated ERα/β-heterodimer specificity using BRET and reporter assays and showed that 1 µM cosmosiin and 10 µM angolensin are specific to ERα/β-heterodimers (Fig. 3).

Using ERα/β-heterodimer selective compounds at specific concentrations, we are able to show that the ERα/β-heterodimer is growth inhibitory. These compounds inhibit cell proliferation in HC11 and PC3 cells which co-express ERα and ERβ. Inhibition of cell growth (Fig. 6) and migration (Fig. 5) due to 1 µM cosmosiin and 10 µM angolensin is ablated with treatment of ICI 182,780 or the silencing of either ERα or ERβ in PC3-shERα and PC3-shERβ, respectively. These compounds, however, did not have an effect on ER-negative MDA-MB-231 and ERα-negative/ERβ-positive DU-145 cells, further supporting that the growth inhibitory effects observed with these compounds were dependent on expression of both ERα and ERβ. While these compounds appear to have little or no effect on ERα/α homodimerization and transcriptional activation in HEK293 BRET and ERE-luciferase assays employing exogenous ERs (Fig. 3), treatment of breast and prostate cancer cells expressing ERα at a much higher level than ERβ (PC3-shERβ) results in ERα-dependent growth increases (Fig. 6E). This result is in agreement with the common theme that ERα is a major growth driver, and it also implicates the dependence of these compounds' growth effects on the relative expression ratio of ERα∶ERβ, as these compounds ablate growth increases in PC3 and HC11, in which ERα and ERβ expression levels are relatively similar and heterodimerization may be favored [31]. Taken together, these data suggest that the ratio of ERα∶ERβ in the same tumor cells is extremely important for physiological effects of these compounds. While the data presented herein provide initial evidence for a growth-inhibitory function of the ERα/β heterodimer, identification of higher affinity compounds with greater ERα/β heterodimer selectivity will be needed to validate our findings since both compounds are weak agonists, and cosmosiin at 10 µM appears to have off-target effects.

Compounds exhibiting ERα/β heterodimer-selectivity may have therapeutic or preventive efficacy in hormone-dependent diseases. A recent study shows that the tamoxifen metabolite endoxifen is capable of degrading ERα [45], stabilizing ERβ, and inducing ERα/β heterodimerization in a concentration dependent manner [46]. Tamoxifen is a widely-utilized FDA-approved breast cancer treatment and prevention drug. This finding suggests that tamoxifen's cancer preventive effects may be mediated by stimulation of ERα/β heterodimer formation. The possibility is supported by the fact that both ERs are expressed in normal mammary epithelial cells [4]. Similarly, naturally-occurring estrogen-like compounds such as phytoestrogens, a group of plant-derived compounds with estrogenic and/or antiestrogenic activities hold promise for action as preventive or therapeutic ER-regulators via their abilities to mediate estrogenic responses tissue-specifically. Indeed, consumption of soy phytoestrogens has been correlated with decreased breast cancer risk [47], although these data remain somewhat controversial [48]. Furthermore, consumption of genistein [49], resveratrol [50], and soy [51] has been inversely correlated with prostate cancer risk. Although these compounds may stimulate the proliferative action of ERα when ERβ is lost in tumors, they may have preventative effects under normal physiological conditions when both ERs are expressed.

Furthermore, our examination of nuclear co-localization of ERα and ERβ within the same tumor cell using the AQUA® technology (Fig. 7) support that ERα/β heterodimerization could potentially occur within tumor cells. Prior to these studies, the co-localization of ERα and ERβ within the same cell had not been examined. The punctate staining pattern suggests that ERα and ERβ are co-localized on DNA, and therefore may be transcriptionally active in these cells as ERα/β heterodimers. Furthermore, AQUA® analysis showed that the ERα∶ERβ ratio is higher in malignant states compared to benign tissue samples, in agreement with the finding that ERβ levels often decrease in malignant breast cancers [52]. The growth inhibitory effects of ERα/β heterodimers might due to their activation of different target genes from their respective homodimers. Recently, global ChIP-Seq analyses of ERα and ERβ target genes show that perfectly or imperfectly palindromic EREs are preferential binding sites for ERα/β heterodimers as compared to ERα/α or ERβ/β homodimers which are more flexible in DNA recognition [53]. This is consistent with other reports that ERα/β heterodimers might regulate distinct genes [32], [33]. The ERα/β heterodimer-selective ligands identified in this study will allow identification of heterodimer target genes in cells co-expressing ERα and ERβ (e.g. PC3). While our findings implicate the ERα/β heterodimer as a putative preventative and therapeutic target for hormone-responsive cancers, this example highlights the imminent need to decipher the role these heterodimers in breast and prostate cancers.

In conclusion, these data provide a proof-of-principle that ERα/β heterodimer-selective ligands can inhibit cell growth and migration in ERα/ERβ-positive cells such as PC3 and HC11 when ERα and ERβ are expressed at similar levels. We also found that the compounds' growth effects depend on the relative expression levels of ERα and ERβ. Upon knockdown of ERβ in PC3 cells, cosmosiin increases PC3 cell growth in an ERα-dependent manner. Thus, more heterodimer selective ligands need to be identified to clarify whether the heterodimer-selective ligands become growth stimulatory when ERβ expression is lost in human tumors. Although more studies are needed to demonstrate the ERα/β heterodimer as a therapeutic target, the concept of inducing ERβ to pair with ERα, thus antagonizing ERα's proliferative function, is distinct from existing breast cancer therapeutic strategies of targeting ERα alone. We also suggest that the relative ERα and ERβ expression levels in patient tumors should be carefully evaluated to better understand the ER-targeted drugs' therapeutic performance, as many of these drugs have not been evaluated for their dimer selectivity, and ERβ expression in patient tumors is not routinely evaluated.

## Materials and Methods

### High Throughput Screening Methods

All primary and secondary screens were performed at the University of Wisconsin Carbone Cancer Center (UWCCC) Keck Small Molecule Screening Facility (SMSF). Ten thousand T47D-KBLuc cells [54] were seeded into 384-well plates and allowed to attach overnight. The next day, 0.5 µl of 1 mM compound was added to a final concentration of 10 µM using an automated robotic system (Beckman Biomek FX). 10 nM E2 and 1% (0.5 µl) DMSO were used as positive and negative controls, respectively. Cells were incubated with compound for 18 hrs at 37°C in 5% CO_2_ in a cell culture incubator. On day 3, media were removed, and 25 µl lysis buffer (Promega, cat# E2661) was added to each well using the robot. Cells were allowed to lyse for 10 min with constant agitation, and lysis was confirmed by microscopically viewing a clear-bottom 384-well plate maintained in parallel under identical conditions. 25 µl luciferase substrate (Promega, Cat# E2620) was then added, mixed for 30 seconds, and luciferase emission was immediately detected on a Tecan Safire 2 plate reader at 0.1 seconds per well. Counter-screening was performed in a similar fashion in the presence and absence of the ER antagonist ICI 182,780. Secondary Bioluminescence Resonance Energy Transfer (BRET) screening was performed in transiently transfected HEK293 cells (ATCC, CRL-1573). DNA encoding BRET fusions were transfected as described in [34]. Following 24 hours of protein expression after transfection, cells were trypsinized and counted using a Nexcelcom Cellometer, and cell viability was determined to be >95% in each condition. Cells were seeded at 11,000 cells per well of 384-well white-walled white-bottom plates in 40 µL PBS. 0.2 µL of 1 mM library compound was then added to each well using the Biomek FX Robot such that the final concentration per well was 5 µM. Cell suspensions were incubated with library compounds for 1 hour in a dark cabinet at room temperature, at which point 10 µL of the Renilla Luciferase (RLuc) substrate coelenterazine h was added to a final concentration of 5 µM. Plates were then gently shaken on a plate shaker for 10 seconds at 300 rpm, and RLuc emission was read at 460 nm followed immediately by YFP emission at 535 nm at 0.1 second per wavelength read per well. Each RLuc and YFP emission measurement was taken consecutively per well before moving to the next well. Emission values were used to calculate the BRET ratio as described in [34]. Additional details for BRET screening were described in [35].

### In vivo BRET assays to monitor ER dimer formation in living cells

HEK293 cells (ATCC, CRL-1573) were either transfected with a single BRET fusion plasmid (pCMX-ERα-RLuc or pCMX-RLuc-ERβ) or co-transfected with RLuc and YFP BRET fusions (pCMX-ERα-RLuc+pCMX-YFP-ERβ for ERα/ERβ heterodimers; pCMX-ERα-RLuc+pCMX-ERα-YFP for ERα homodimers; or pCMX-RLuc-ERβ+pCMX-YFP-ERβ for ERβ homodimers) [34]. “Empty” expression vector pCMX-pL2 was used to keep the total amount of transfected DNA constant. 24 hr post-transfection, cells were trypsinized, counted, and resuspended in PBS in quadruplicate at ∼50,000 cells per well of a 96-well white-bottom microplate. Cells were incubated with ligands for 1 hour. Coelenterazine h (Promega, Madison, WI) was added in PBS at a final concentration of 5 µM, and 460 nm and 530 nm emission detection measurements were immediately taken at 0.1 second per wavelength read per well on a Perkin Elmer Victor 3-V plate reader.

### Immunofluorescence Staining

Deparaffinization and heat induced epitope retrieval were performed simultaneously using the Lab Vision PT module (Thermo Fisher Scientific, Fremont, CA) with Lab Vision citrate buffer pH 8.0 at 98°C for 20 minutes. All staining was performed at room temperature using the Lab Vision 360 automated staining system. Endogenous peroxidase was blocked for 5 minutes with Peroxidazed-1 (Cat.No. PX968, Biocare Medical). Non-specific protein binding was eliminated via a 60 minute block with Biocare Medical Sniper, and non-specific avidin was blocked using Biocare Medical Avidin Biotin kit, incubating 15 minutes. DaVinci Green Antibody Diluent (Cat.No. PD900L, Biocare Medical) was used for antibody dilution. Breast TMA BR2082 containing 208 cores was purchased from US Biomax Inc. (http://www.biomax.us/tissue-arrays/Breast/BR2082). ERα was detected using ERα rabbit mAb SP1 (1∶50, 1 hr) (Thermo Fisher) and visualized with goat anti-rabbit conjugated with Alexa Fluor 555 (Invitrogen) secondary antibody. ERβ was detected with mouse mAb 14C8 (Abcam,1∶1600, 1 hr) and visualized with Alexa Fluor 647 conjugated Tyramide Signal Amplification system (Invitrogen), which included biotinylated goat anti-mouse immunoglobulin, streptavidin-horseradish peroxidase and Alexa Fluor 647-Tyramide. Breast epithelial nuclei were masked using ProLong Gold Antifade Reagent with DAPI mounting medium (Invitrogen).

### Automated Image Acquisition

Automated image capture was performed by the HistoRx PM-2000 using the AQUAsition software package (New Haven, CT). High-resolution (2048_2048 pixel, 7.4 mm), 8-bit grayscale digital images are obtained for each area of interest resulting in 256 discrete intensity values per pixel of an acquired image [55]. The breast epithelial nuclear compartment was defined with DAPI (blue). The target markers (ERα and ERβ) were visualized with Alexa Fluor 555 (green) and 647 (red), respectively.

### AQUA ® Score Generation

Since the distributions of the original AQUA® scores exhibited deviation from the normal distribution, we took the natural log transformation of the original scores and then performed two sample t-tests for pairwise comparisons among different samples. Results from these tests were consistent with a Wilcoxon rank sum test on the original scores. Images were evaluated before scoring. Histospots showing <5% tumor area, tissue folding, too much debris, and those that were out of focus were disqualified from scoring. Nuclear AQUA® scores for ERα and ERβ for each histospot were generated based on the unsupervised pixel-cased clustering algorithm for optimal image segmentation for use in the pixel-based locale assignment for compartmentalization of expression algorithm as described previously [56]. Pixels that could not accurately be assigned to a compartment were discarded. The data were saved and subsequently expressed as the average signal intensity per unit of compartment area. All the signals in each compartment were then added. The AQUA® score is expressed as target signal intensity divided by the compartment pixel area and is expressed on a scale of 0 to 33333 (AQUA_2.0, HistoRx). The resultant AQUA® score is continuous and directly proportional to the number of molecules per unit area.

Additional descriptions of cell culture, TMA and experimental procedures can be found in **Methods S1**.

## Supporting Information

Figure S1 **Caspase 3/7 apoptosis assays showed that cosmosiin and angolensin exhibited no apoptotic effect via caspases 3 or 7 in PC3 cells at 96 hours.** Cosmosiin (A) and angolensin (B) modestly increase apoptosis through this pathway in PC3 cells to a statistically non-significant level compared to the strong apoptotic inducer cisplatin, which served as a positive control. Statistical analysis method: Students T-Tests. Error bars represent standard deviations from the mean of triplicate samples.
(TIF)

Figure S2 **MTT assays showing the effect of 10 nM 17β-estradiol in ERα,β-positive PC3 cells and variants of these cells in which ERα has been silenced (PC3-shERα) or ERβ has been silenced (PC3-shERβ).** E2 has no effect on the proliferation of PC3 cells (A) or PC3-shERα cells (B); however, the silencing of ERβ in this cell line allows E2 to increase cellular growth (C, left panel) by binding to ERα, since the presence of the antagonists ICI 182,780 (C, middle panel) and MPP Dihydrochloride (C, right panel) ablates this increase. Statistical analysis method: Students T-Test; * indicates p<0.05. Error bars represent standard deviations from the mean of triplicate samples.
(TIF)

Figure S3 **Cosmosiin at 1 µM and angolensin at 10 µM inhibited the growth of ERα/ERβ positive HC11 cells in an ER-dependent manner.** Cosmosiin (A) and angolensin (B) had no cytotoxic effects at all tested concentrations. The growth inhibitory effects of cosmosiin at 1 µM (C) and angolensin at 10 µM (D) were ablated by pure ER antagonist ICI 182,780, suggesting the growth inhibitory effects are ER-dependent. These decreases due to 10 µM cosmosiin are ER-independent since they are retained in the presence of the antagonist ICI 182,780 (C). Statistical analysis method: Students T-Test; * indicates p<0.05. Error bars represent standard deviations from the mean of triplicate samples.
(TIF)

Figure S4 **Neither cosmosiin (A) nor angolensin (B) influenced the growth of ER-negative MDA-MB-231breast cancer cells.** Error bars represent standard deviations from the mean of triplicate samples.
(TIF)

Figure S5 **Neither cosmosiin (A) nor angolensin (B) influenced the growth of ERα-negative, ERβ-positive DU-145 prostate cancer cells.** Error bars represent standard deviations from the mean of triplicate samples.
(TIF)

Methods S1 **Supplemental Methods file.**
(DOC)

## References

[1] Heldring N, Pike A, Andersson S, Matthews J, Cheng G (2007). Estrogen receptors: how do they signal and what are their targets.. Physiol Rev.

[2] Deroo BJ, Korach KS (2006). Estrogen receptors and human disease.. J Clin Invest.

[3] Nilsson S, Gustafsson JA (2011). Estrogen receptors: therapies targeted to receptor subtypes.. Clin Pharmacol Ther.

[4] Shoker BS, Jarvis C, Sibson DR, Walker C, Sloane JP (1999). Oestrogen receptor expression in the normal and pre-cancerous breast.. J Pathol.

[5] Hewitt SC, Harrell JC, Korach KS (2005). Lessons in estrogen biology from knockout and transgenic animals.. Annu Rev Physiol.

[6] Forster C, Makela S, Warri A, Kietz S, Becker D (2002). Involvement of estrogen receptor beta in terminal differentiation of mammary gland epithelium.. Proc Natl Acad Sci U S A.

[7] McPherson SJ, Ellem SJ, Patchev V, Fritzemeier KH, Risbridger GP (2006). The role of Eralpha and ERbeta in the prostate: insights from genetic models and isoform-selective ligands.. Ernst Schering Found Symp Proc.

[8] Imamov O, Morani A, Shim GJ, Omoto Y, Thulin-Andersson C (2004). Estrogen receptor beta regulates epithelial cellular differentiation in the mouse ventral prostate.. Proc Natl Acad Sci U S A.

[9] Ricke WA, McPherson SJ, Bianco JJ, Cunha GR, Wang Y (2008). Prostatic hormonal carcinogenesis is mediated by in situ estrogen production and estrogen receptor alpha signaling.. FASEB J.

[10] Krege JH, Hodgin JB, Couse JF, Enmark E, Warner M (1998). Generation and reproductive phenotypes of mice lacking estrogen receptor beta.. Proc Natl Acad Sci U S A.

[11] Jarvinen TA, Pelto-Huikko M, Holli K, Isola J (2000). Estrogen receptor beta is coexpressed with ERalpha and PR and associated with nodal status, grade, and proliferation rate in breast cancer.. Am J Pathol.

[12] Zhu X, Leav I, Leung YK, Wu M, Liu Q (2004). Dynamic regulation of estrogen receptor-beta expression by DNA methylation during prostate cancer development and metastasis.. Am J Pathol.

[13] Chang EC, Frasor J, Komm B, Katzenellenbogen BS (2006). Impact of estrogen receptor beta on gene networks regulated by estrogen receptor alpha in breast cancer cells.. Endocrinology.

[14] Helguero LA, Faulds MH, Gustafsson JA, Haldosen LA (2005). Estrogen receptors alfa (ERalpha) and beta (ERbeta) differentially regulate proliferation and apoptosis of the normal murine mammary epithelial cell line HC11.. Oncogene.

[15] Pettersson K, Delaunay F, Gustafsson JA (2000). Estrogen receptor beta acts as a dominant regulator of estrogen signaling.. Oncogene.

[16] Lazennec G, Bresson D, Lucas A, Chauveau C, Vignon F (2001). ER beta inhibits proliferation and invasion of breast cancer cells.. Endocrinology.

[17] Murphy LC, Peng B, Lewis A, Davie JR, Leygue E (2005). Inducible upregulation of oestrogen receptor-beta1 affects oestrogen and tamoxifen responsiveness in MCF7 human breast cancer cells.. J Mol Endocrinol.

[18] Rousseau C, Nichol JN, Pettersson F, Couture MC, Miller WH (2004). ERbeta sensitizes breast cancer cells to retinoic acid: evidence of transcriptional crosstalk.. Mol Cancer Res.

[19] Strom A, Hartman J, Foster JS, Kietz S, Wimalasena J (2004). Estrogen receptor beta inhibits 17beta-estradiol-stimulated proliferation of the breast cancer cell line T47D.. Proc Natl Acad Sci U S A.

[20] Omoto Y, Inoue S, Ogawa S, Toyama T, Yamashita H (2001). Clinical value of the wild-type estrogen receptor beta expression in breast cancer.. Cancer Lett.

[21] Skliris GP, Carder PJ, Lansdown MR, Speirs V (2001). Immunohistochemical detection of ERbeta in breast cancer: towards more detailed receptor profiling?. Br J Cancer.

[22] Roger P, Sahla ME, Makela S, Gustafsson JA, Baldet P (2001). Decreased expression of estrogen receptor beta protein in proliferative preinvasive mammary tumors.. Cancer Res.

[23] Iwao K, Miyoshi Y, Egawa C, Ikeda N, Tsukamoto F (2000). Quantitative analysis of estrogen receptor-alpha and -beta messenger RNA expression in breast carcinoma by real-time polymerase chain reaction.. Cancer.

[24] Iwao K, Miyoshi Y, Egawa C, Ikeda N, Noguchi S (2000). Quantitative analysis of estrogen receptor-beta mRNA and its variants in human breast cancers.. Int J Cancer.

[25] Paruthiyil S, Parmar H, Kerekatte V, Cunha GR, Firestone GL (2004). Estrogen receptor beta inhibits human breast cancer cell proliferation and tumor formation by causing a G2 cell cycle arrest.. Cancer Res.

[26] Williams C, Edvardsson K, Lewandowski SA, Strom A, Gustafsson JA (2008). A genome-wide study of the repressive effects of estrogen receptor beta on estrogen receptor alpha signaling in breast cancer cells.. Oncogene.

[27] Frasor J, Chang EC, Komm B, Lin CY, Vega VB (2006). Gene expression preferentially regulated by tamoxifen in breast cancer cells and correlations with clinical outcome.. Cancer Res.

[28] Chang EC, Charn TH, Park SH, Helferich WG, Komm B (2008). Estrogen Receptors alpha and beta as determinants of gene expression: influence of ligand, dose, and chromatin binding.. Mol Endocrinol.

[29] Saji S, Hirose M, Toi M (2005). Clinical significance of estrogen receptor beta in breast cancer.. Cancer Chemother Pharmacol.

[30] Lindberg MK, Moverare S, Skrtic S, Gao H, Dahlman-Wright K (2003). Estrogen receptor (ER)-beta reduces ERalpha-regulated gene transcription, supporting a “ying yang” relationship between ERalpha and ERbeta in mice.. Mol Endocrinol.

[31] Cowley SM, Hoare S, Mosselman S, Parker MG (1997). Estrogen receptors alpha and beta form heterodimers on DNA.. J Biol Chem.

[32] Pettersson K, Grandien K, Kuiper GG, Gustafsson JA (1997). Mouse estrogen receptor beta forms estrogen response element-binding heterodimers with estrogen receptor alpha.. Mol Endocrinol.

[33] Tremblay GB, Tremblay A, Labrie F, Giguere V (1999). Dominant activity of activation function 1 (AF-1) and differential stoichiometric requirements for AF-1 and -2 in the estrogen receptor alpha-beta heterodimeric complex.. Mol Cell Biol.

[34] Powell E, Xu W (2008). Intermolecular interactions identify ligand-selective activity of estrogen receptor alpha/beta dimers.. Proc Natl Acad Sci U S A.

[35] Powell E, Huang SX, Xu Y, Rajski SR, Wang Y (2010). Identification and Characterization of a Novel Estrogenic Ligand Actinopolymorphol A.. Biochem Pharmacol.

[36] Lau KM, LaSpina M, Long J, Ho SM (2000). Expression of estrogen receptor (ER)-alpha and ER-beta in normal and malignant prostatic epithelial cells: regulation by methylation and involvement in growth regulation.. Cancer Res.

[37] Mosmann T (1983). Rapid colorimetric assay for cellular growth and survival: application to proliferation and cytotoxicity assays.. J Immunol Methods.

[38] Monroe DG, Secreto FJ, Subramaniam M, Getz BJ, Khosla S (2005). Estrogen receptor alpha and beta heterodimers exert unique effects on estrogen- and tamoxifen-dependent gene expression in human U2OS osteosarcoma cells.. Mol Endocrinol.

[39] Stossi F, Barnett DH, Frasor J, Komm B, Lyttle CR (2004). Transcriptional profiling of estrogen-regulated gene expression via estrogen receptor (ER) alpha or ERbeta in human osteosarcoma cells: distinct and common target genes for these receptors.. Endocrinology.

[40] Aherne SA, O'Brien NM (2002). Dietary flavonols: chemistry, food content, and metabolism.. Nutrition.

[41] Heinonen SM, Wahala K, Adlercreutz H (2004). Identification of urinary metabolites of the red clover isoflavones formononetin and biochanin A in human subjects.. J Agric Food Chem.

[42] Pfitscher A, Reiter E, Jungbauer A (2008). Receptor binding and transactivation activities of red clover isoflavones and their metabolites.. J Steroid Biochem Mol Biol.

[43] Fuchs J, Milbradt R (1993). Skin anti-inflammatory activity of apigenin-7-glucoside in rats.. Arzneimittelforschung.

[44] Wang HK, Xia Y, Yang ZY, Natschke SL, Lee KH (1998). Recent advances in the discovery and development of flavonoids and their analogues as antitumor and anti-HIV agents.. Adv Exp Med Biol.

[45] Wu X, Hawse JR, Subramaniam M, Goetz MP, Ingle JN (2009). The tamoxifen metabolite, endoxifen, is a potent antiestrogen that targets estrogen receptor alpha for degradation in breast cancer cells.. Cancer Res.

[46] Wu X, Subramaniam M, Grygo SB, Sun Z, Negron V (2011). Estrogen receptor-beta sensitizes breast cancer cells to the anti-estrogenic actions of endoxifen.. Breast Cancer Res.

[47] Peeters PH, Keinan-Boker L, van der Schouw YT, Grobbee DE (2003). Phytoestrogens and breast cancer risk. Review of the epidemiological evidence.. Breast Cancer Res Treat.

[48] Ju YH, Allred KF, Allred CD, Helferich WG (2006). Genistein stimulates growth of human breast cancer cells in a novel, postmenopausal animal model, with low plasma estradiol concentrations.. Carcinogenesis.

[49] Mentor-Marcel R, Lamartiniere CA, Eltoum IA, Greenberg NM, Elgavish A (2005). Dietary genistein improves survival and reduces expression of osteopontin in the prostate of transgenic mice with prostatic adenocarcinoma (TRAMP).. J Nutr.

[50] Jones SB, DePrimo SE, Whitfield ML, Brooks JD (2005). Resveratrol-induced gene expression profiles in human prostate cancer cells.. Cancer Epidemiol Biomarkers Prev.

[51] Lee MM, Gomez SL, Chang JS, Wey M, Wang RT (2003). Soy and isoflavone consumption in relation to prostate cancer risk in China.. Cancer Epidemiol Biomarkers Prev.

[52] Sugiura H, Toyama T, Hara Y, Zhang Z, Kobayashi S (2007). Expression of estrogen receptor beta wild-type and its variant ERbetacx/beta2 is correlated with better prognosis in breast cancer.. Jpn J Clin Oncol.

[53] Grober OM, Mutarelli M, Giurato G, Ravo M, Cicatiello L (2011). Global analysis of estrogen receptor beta binding to breast cancer cell genome reveals an extensive interplay with estrogen receptor alpha for target gene regulation.. BMC Genomics.

[54] Wilson VS, Bobseine K, Gray LE (2004). Development and characterization of a cell line that stably expresses an estrogen-responsive luciferase reporter for the detection of estrogen receptor agonist and antagonists.. Toxicol Sci.

[55] Warren M, Twohig M, Pier T, Eickhoff J, Lin CY (2009). Protein expression of matriptase and its cognate inhibitor HAI-1 in human prostate cancer: a tissue microarray and automated quantitative analysis.. Appl Immunohistochem Mol Morphol.

[56] Gustavson MD, Bourke-Martin B, Reilly DM, Cregger M, Williams C (2009). Development of an unsupervised pixel-based clustering algorithm for compartmentalization of immunohistochemical expression using Automated QUantitative Analysis.. Appl Immunohistochem Mol Morphol.

