# Supplementary figures and images for: Identification of Estrogen Receptor Dimer Selective Ligands Reveals Growth-Inhibitory Effects on Cells That Co-Express ERα and ERβ
Source: PLoS One. 2012 Feb 7;7(2):e30993. doi: 10.1371/journal.pone.0030993 (PMC3274540; doi:10.1371/journal.pone.0030993)

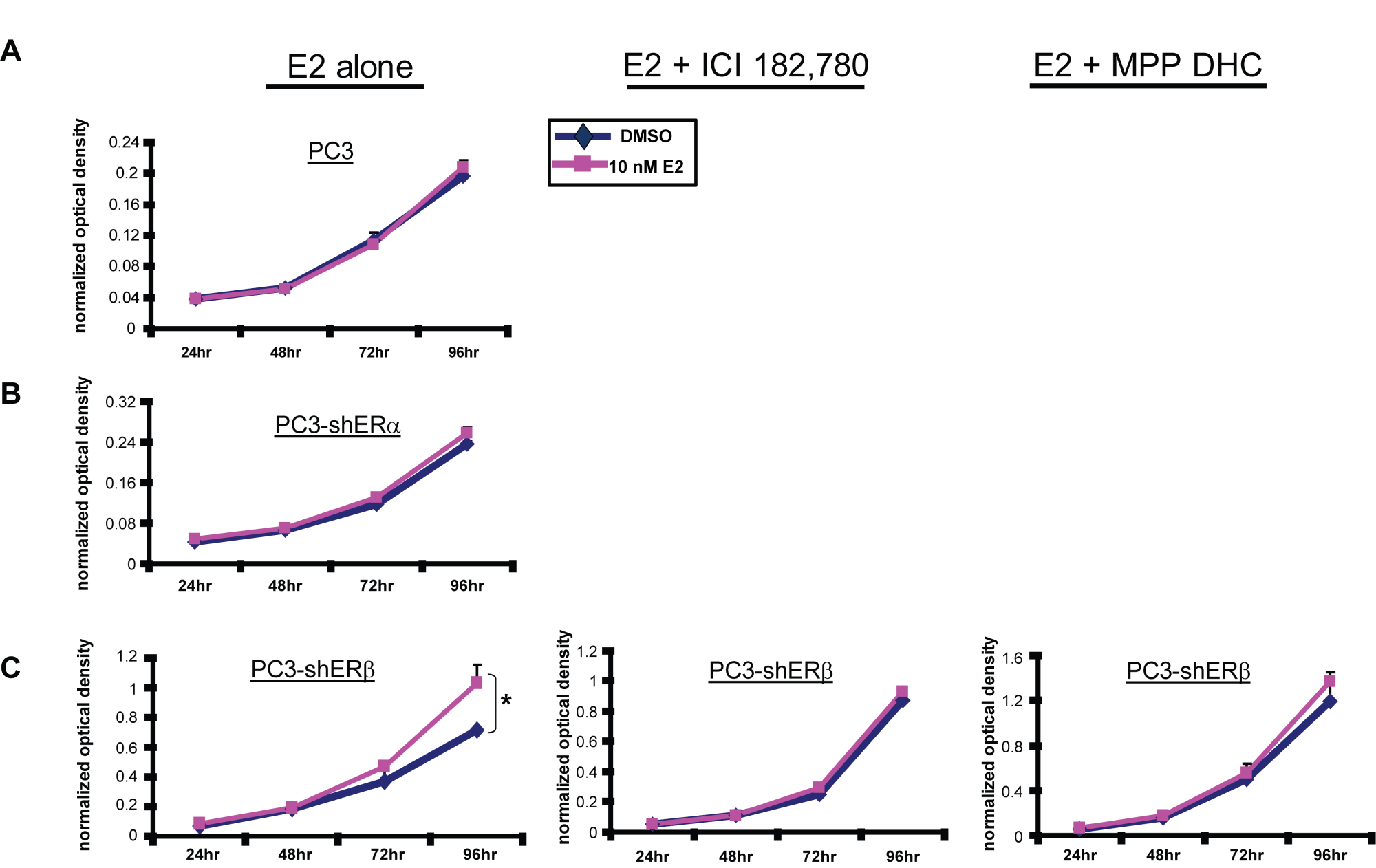

Supplement: Figure S2 — MTT assays showing the effect of 10 nM 17β-estradiol in ERα,β-positive PC3 cells and variants of these cells in which ERα has been silenced (PC3-shERα) or ERβ has been silenced (PC3-shERβ). E2 has no effect on the proliferation of PC3 cells (A) or PC3-shERα cells (B); however, the silencing of ERβ in this cell line allows E2 to increase cellular growth (C, left panel) by binding to ERα, since the presence of the antagonists ICI 182,780 (C, middle panel) and MPP Dihydrochloride (C, right panel) ablates this increase. Statistical analysis method: Students T-Test; * indicates p<0.05. Error bars represent standard deviations from the mean of triplicate samples. (TIF) [file pone.0030993.s002.tif]

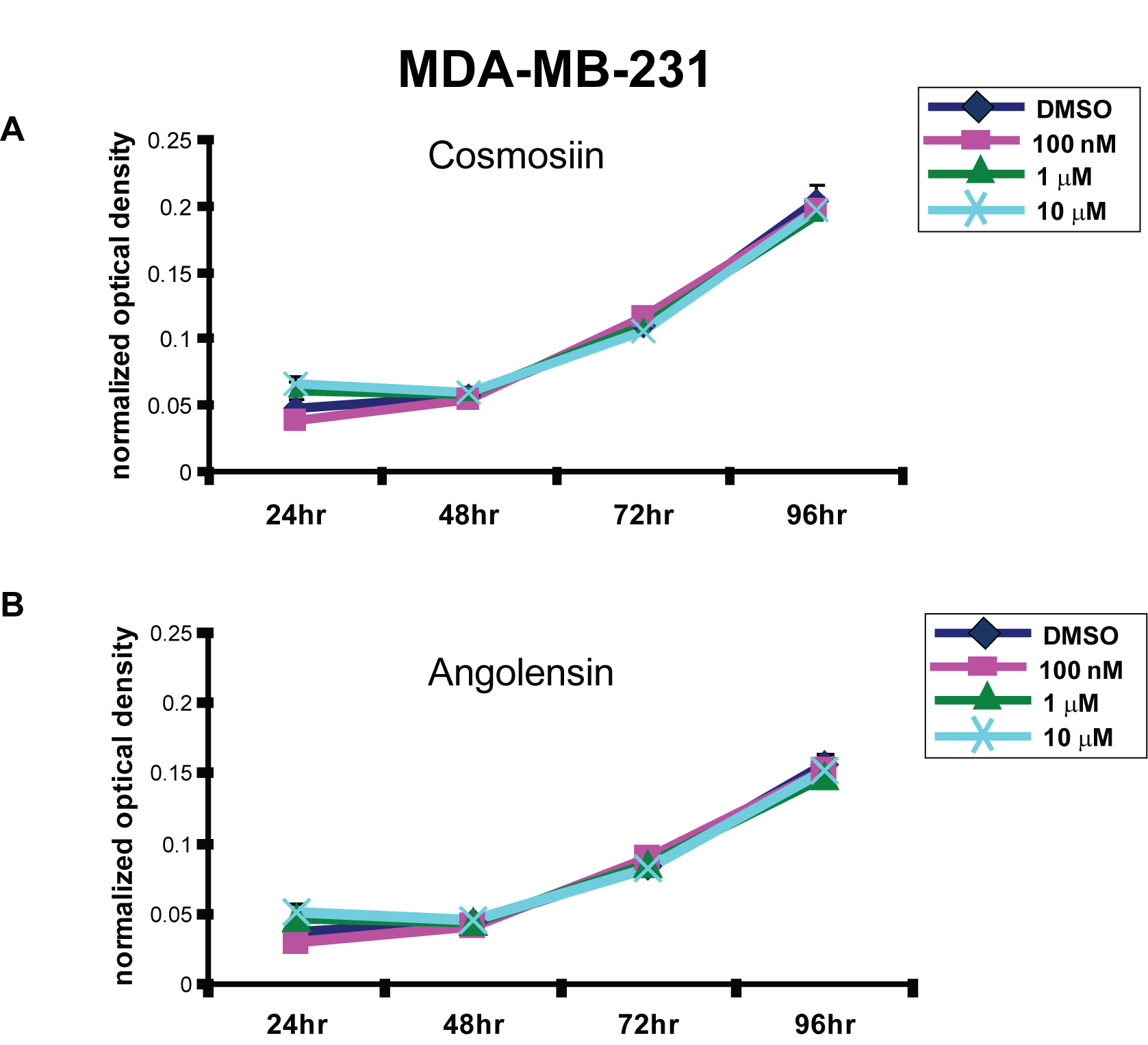

Supplement: Figure S4 — Neither cosmosiin (A) nor angolensin (B) influenced the growth of ER-negative MDA-MB-231breast cancer cells. Error bars represent standard deviations from the mean of triplicate samples. (TIF) [file pone.0030993.s004.tif]

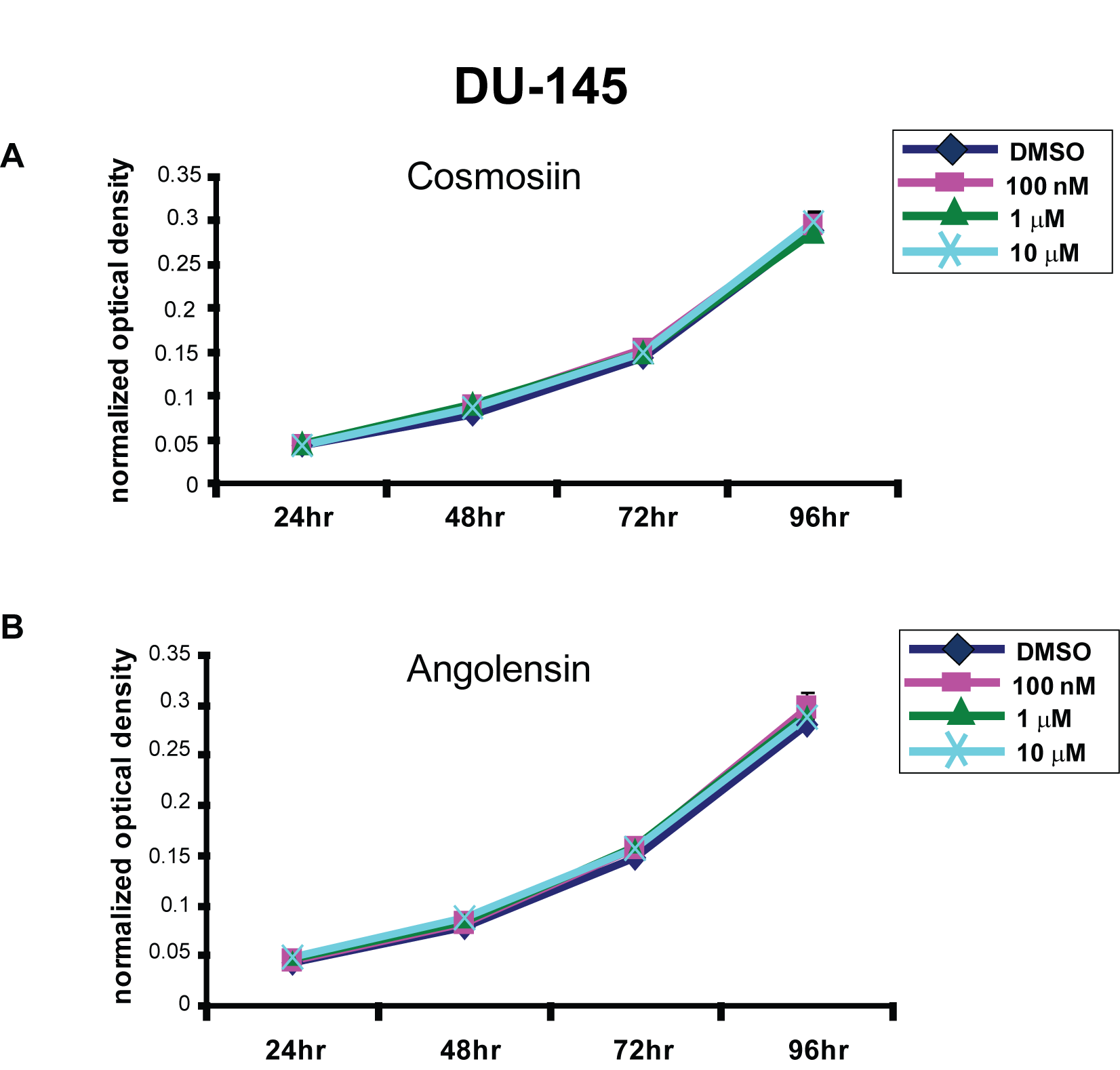

Supplement: Figure S5 — Neither cosmosiin (A) nor angolensin (B) influenced the growth of ERα-negative, ERβ-positive DU-145 prostate cancer cells. Error bars represent standard deviations from the mean of triplicate samples. (TIF) [file pone.0030993.s005.tif]
