# Supplementary material for: Identification of Estrogen Receptor Dimer Selective Ligands Reveals Growth-Inhibitory Effects on Cells That Co-Express ERα and ERβ
Source: PLoS One. 2012 Feb 7;7(2):e30993. doi: 10.1371/journal.pone.0030993 (PMC3274540; doi:10.1371/journal.pone.0030993)
Supplement: Methods S1 — Supplemental Methods file. (DOC) [file pone.0030993.s006.doc]

**Supplemental Methods:**

**Identification of Estrogen Receptor dimer selective ligands reveals growth-inhibitory effects on cells that co-express ERα and ERβ**

Emily Powell, Erin Shanle, Ashley Brinkman, Jun Li, Sunduz Keles, Kari Wisinski, Wei Huang, Wei Xu

**Inventory of Supplementary information**

Supplementary experimental procedures

Cell culture

Breast TMA BR2082

Fluorescence Polarization Competition Ligand Binding assays

Luciferase reporter assays

MTT assays for measuring cellular metabolic activity

Cell Counting and Viability Assays

Wound healing assays

Transwell cell invasion assays

Apoptosis assays

Western blotting

Construction of ERα and ERβ shRNA expression plasmids and PC3shER stable cell lines

**SUPPLEMENTARY EXPERIMENTAL PROCEDURES**

**Cell culture**

MDA-MB-231 breast cancer cells were purchased from ATCC (cat. no. HTB-26) and were maintained in DMEM + 10% FBS. PC3 and DU-145 human prostate cancer cells were kindly provided by the laboratory of Dr. Douglas McNeel (Department of Medicine, UW-Madison) and were maintained in DMEM + 10% FBS. HC11 normal mouse mammary epithelial cells were kindly provided by the laboratory of Dr. Caroline Alexander (Department of Oncology, UW-Madison) and were maintained in RPMI1640 + 10 ng/mL EGF, 5 µg/ml insulin, and 10% FBS.

**Breast TMA BR2082**

The breast TMA BR2082 was purchased from US Biomax Inc. (<http://www.biomax.us/tissue-arrays/Breast/BR2082>). This TMA contains 208 cores with 32 metastasis, 116 malignant tumor (68 invasive ductal carcinoma, 22 each of lobular carcinoma and intraductal carcinoma), 8 benign tumor, 16 hyperplasia, 4 each of squamous cell carcinoma and lobular carcinoma in situ, 16 hyperplasia and inflammation (NAT), 10 benign tumor, 6 normal tissues.

**Fluorescence Polarization Competition Ligand Binding assays**

Affinity of ligands for ERα and ERβ was measured using Estrogen Receptor Competitor Assays from Invitrogen (PanVera) (Part # P2614, P2698 for ERα; Part # P2615, P2700 for ERβ) according to the manufacturer’s instructions. Using purified ERα and ERβ provided in the kit, serial dilutions of test compounds were prepared from 2 mM to 20 pM by preparing 1:10 dilutions in provided screening buffer. The concentration of DMSO was kept below 1%. Compounds were diluted 2-fold in the final reaction with a mixture of 30 nM purified ERα, 2 nM Fluormone, and screening buffer such that the final concentration of test ligand were serially diluted 1 mM to 10 pM, the final concentration of ERα was 15 nM, and the final concentration of Fluormone was 1 nM. Test ligands were prepared in a similar fashion for ERβ using a final concentration of 10 nM ERβ. This mixture was incubated in the dark at room temperature for 2 hours, and polarization values were read in individual glass tubes using a Beacon 2000 instrument.

**Luciferase reporter assays**

HEK293 cells were transfected in batches in 48-well plates using 2.5 ng of each indicated ER and 50 ng tk-ERE-luc vector per well. After allowing 48 hours for protein expression and incubating with the indicated ligands for 24 hours, cells were lysed, and firefly luciferase emission was detected upon addition of the firefly luciferase substrate (Promega) on a PerkinElmer Victor 3-V plate reader using a luminescence detection setting. β-gal was analyzed using the Tropix β-galactosidase detection kit (Tropix), and emission was detected on a PerkinElmer Victor 3-V plate reader using a luminescence detection setting. Luciferase counts were normalized to β-gal counts in each well.

**MTT assays for measuring cellular metabolic activity**

These assays were performed in a variety of cell types following the same protocol. Cells were seeded to a confluency of ~10% on Day 1 in phenol red free DMEM supplemented with 5% FBS stripped 6 times with charcoal and dextran (SFS) in 48-well plates. Cells were allowed to attach overnight, and on Day 2 the appropriate dilution of DMSO vehicle, 10 nM E2, or lead compound was added with a final DMSO concentration of 0.1%. Four consecutive time points were then harvested on Days 3, 4, 5, and 6 (24 hours, 48 hours, 72 hours, and 96 hours post-treatment), and ligands were refreshed every 48 hours by replacing the media. Each time point was harvested by adding MTT to a final concentration of 500 µg/ml and incubating for 30 minutes at 37^o^C, 5% CO_2_ in humidified air. The media + MTT solution was removed from each well with suction and 50 µL of DMSO was added to each well, incubated at room temperature with shaking to solublize the purple formazan crystals, and transferred to a clear flat-bottom 96-well plate. The plate was then read at 595 nm for formazan absorbance and 650 nm for background absorbance, and these values were normalized by subtraction. Each condition was performed in triplicate.

**Cell Counting and Viability Assays**

1 x10^5^ cells were seeded onto 6 cm plates in phenol red free DMEM + 5% SFS and allowed to attach overnight. The next day, media was replaced with media containing the indicated concentration of ligands or 0.1% DMSO, and the total amount of DMSO per plate was kept constant at 0.1%. Time points were harvested at 24 hours, 48 hours, 72 hours, and 96 hours by trypsinizing cells, inactivating the trypsin with DMEM + 10% FBS and transferring to 2 mL eppendorf tubes, and centrifuging at 3000 rpm for 5 minutes. Media + trypsin was then removed from the pelleted cells, and the pellet was resuspended in 200 µL PBS + 200 µL of a 1:1 dilution of Trypan blue and PBS. Cells were then loaded onto cell counting chambers (18 µL per chamber side) in duplicate and each side was read in triplicate using the Cellometer. The Cellometer protocol was “initial cell type” with a dilution of 2, and cell viability was measured on each read. The cell number per mL was then extrapolated to total cell number based on the total 400 µL volume.

**Wound healing assays**

PC3 cells were seeded to ~90% confluency (~ 100,000 cells per well) into 24-well plates in 400 µl phenol red free DMEM + 5% FBS. After allowing cells to attach overnight, wounds were scraped into each nearly-confluent monolayer using a p1000 pipette tip. Each well was washed 3 times with PBS and wounds were image captured using an inverted microscope with attached camera using the Leica Application Suite 2.3.4 R2 software. The final PBS wash was then replaced with 400 µL of media containing ligands or 0.1% DMSO. The total amount of DMSO per condition was kept constant at 0.1%. Wounds were then image captured every 24 hours until wounds were completely closed in the vehicle-treated condition.

**Transwell cell migration assays**

PC3 cells were split 1:10 onto 10 cm plates 2 days prior to initiating Transwell assays. Twenty four hours before cell seeding, attached cells were washed with PBS 3 times on their 10 cm plates to remove all traces of FBS, and media was changed to phenol red free, serum-free DMEM 24 hours before cell seeding. For assays involving Transwell coated with matrigel, each well was coated with a 1:5 dilution of matrigel with ice cold sterile PBS, and 40 µL of this slurry was pipetted onto each Transwell insert. The matrigel was allowed to gel 1 hour in the tissue culture incubator. Serum starved PC3 cells were trypsinized from their 10 cm plates (cells were not allowed to exceed ~60% confluency under serum starvation conditions), and trypsin was inactivated with DMEM + 10% FBS. Cells were pelleted at 1000 rpm for 5 minutes, and pellets were washed three times with PBS to remove all traces of PBS. Cells were counted, and 1 x 10^5^ PC3 cells were seeded per insert in 100 µL total volume in triplicate with treatment ligands or 0.1% DMSO. The total amount of DMSO per condition was kept constant at 0.1%. The bottom chambers were plated with DMEM + 10% FBS, which served as the chemoattractant. Cells were incubated in Transwell plates with treatment ligands for 24 hours, at which point media was gently aspirated from top and bottom chambers, and each chamber was washed twice with PBS (200 µL for top chambers, 400 µL for bottom chambers). Calcein AM was diluted to 2 µg/mL in a non-enzymatic cell dissociation solution, and 350 µL was added to the bottom chambers of each well. Transwell inserts were placed on top, ensuring that no bubbles were trapped underneath, and plates were incubated in the cell culture incubator for 30 minutes. Plates were then removed, sides were gently tapped to remove loosened cells, and plates were placed back into the incubator for an additional 30 minutes. Sides were gently tapped again, and 100 µL was transferred to each well of a 96-well solid black microplate in triplicate. Each well was excited at 485 nm and emission was detected at 520 nm on a Perkin Elmer Victor V plate reader.

**Apoptosis assays**

PC3 cells were seeded at 10,000 cells per well of 96-well solid white microplates in phenol red free DMEM + 5% SFS in 100 µL total volume. Cells were seeded in 96-well clear-bottom microplates under parallel conditions to ensure proper cell confluency and condition. Cells were allowed to attach overnight, and the next day media was gently aspirated and replaced with 100 µL of media containing the indicated ligands. The vehicle DMSO was held constant in each condition at 0.1%. Cells were incubated with ligands for time periods of 1 hour, 2 hours, 4 hours, 6 hours, 48 hours, and 72 hours; at the end of each time course, media was removed by turning the plate upside down and gently flicking the wrist, and 50 µL of PBS was added to each well along with 50 µL of Caspase 3/7 Glo Reagent (Promega). Plates were sealed with parafilm and incubated for 1 hour at room temperature in a dark cabinet. Plates were then read on a Perkin Elmer Victor V plate reader using a standard luminescence protocol.

**Western Blotting**

Cells were lysed with Triton X-100 lysis buffer with added protease inhibitors and 50 µg total protein was loaded onto an 8% SDS-PAGE gel. Proteins were transferred to nitrocellulose membranes for 1 hour (for ERα) or 2 hours (for ERβ) at 0.35 amps. Membranes were blocked overnight in 5% nonfat milk (for ERα) or 10% nonfat milk (for ERβ) in PBS-Tween. ERα was detected with a 1:10,000 dilution of Santa Cruz HC-20, and ERβ was detected with a 1:5000 dilution of Santa Cruz H-150. β-actin was detected with a 1:5000 dilution of Sigma A5441.

**Construction of ERα and ERβ shRNA expression plasmids and selection of stable clones**

ERα and ERβ shRNA were directed against DNA sequences, respectively; human ERα, 5’ AAGGTGGGATACGAAAAGACC-3’, and human ERβ, 5’-AAGCCCAAATGTGTTGTGGCC-3’ as previously reported . The palindrome, 60mer oligonucleotides containing above sequences (forward + reverse) were heated and cooled to anneal hairpins, then the annealed nucleotides were ligated with pSUPERIOR vector pre-digested with HindIII and BglII. This mammalian expression vector allows transient transfection of PC3 cells to check knockdown efficiency. After confirming the effectiveness of shRNA in transient knockdown, the shRNA in pSUPERIOR vector were subcloned to pLVTHM lentiviral vectors which encode GFP protein for stable selection as described . The shRNA expressing pLVTHM plasmids were co-transfected with VSVG, PAX2 plasmids to the packaging HEK293T cells. Two days after transfection, lentivirus was harvested to infect PC3 cells. The GFP positive clones were selected to check the efficiency of ERα, ERβ knockdown by western blotting.

1. Pedram, A., Razandi, M., and Levin, E. R. (2006) *Mol Endocrinol* **20**, 1996-2009

2. Wiznerowicz, M., and Trono, D. (2003) *J Virol* **77**, 8957-8961

3. Liu, T., Wu, J., Huang, Q., Hou, Y., Jiang, Z., Zang, S., and Guo, L. (2008) *Shock* **29**, 603-611
